# Relationship between the GABA Pathway and Signaling of Other Regulatory Molecules

**DOI:** 10.3390/ijms251910749

**Published:** 2024-10-06

**Authors:** Katarzyna Kabała, Małgorzata Janicka

**Affiliations:** Department of Plant Molecular Physiology, Faculty of Biological Sciences, University of Wrocław, Kanonia 6/8, 50-328 Wrocław, Poland; katarzyna.kabala@uwr.edu.pl

**Keywords:** GABA, GABA-T, GAD, H_2_O_2_, NO, melatonin, phytohormones, polyamines, ROS, stress conditions

## Abstract

GABA (gamma-aminobutyric acid) is an amino acid whose numerous regulatory functions have been identified in animal organisms. More and more research indicate that in plants, this molecule is also involved in controlling basic growth and development processes. As recent studies have shown, GABA plays an essential role in triggering plant resistance to unfavorable environmental factors, which is particularly important in the era of changing climate. The main sources of GABA in plant cells are glutamic acid, converted in the GABA shunt pathway, and polyamines subjected to oxidative degradation. The action of GABA is often related to the activity of other messengers, including phytohormones, polyamines, NO, H_2_O_2_, or melatonin. GABA can function as an upstream or downstream element in the signaling pathways of other regulators, acting synergistically or antagonistically with them to control cellular processes. Understanding the role of GABA and its interactions with other signaling molecules may be important for developing crop varieties with characteristics that enable adaptation to a changing environment.

## 1. Introduction

GABA (gamma-aminobutyric acid) is a four-carbon non-proteinogenic amino acid found in microorganisms, plants, and animals. The first report confirming the presence of GABA in plants comes from the analysis of biochemical compounds in potato tubers [1]. This amino acid plays an important role in plant functioning, including growth, development, and responses to adverse environmental conditions or pathogen attacks [2,3,4,5,6,7,8]. GABA affects plant growth and mitigates adverse stress-related effects by activating the antioxidant defense system and interacting with other growth regulators and signaling molecules. The process of GABA production in plant cells is regulated by various endogenous factors. On the other hand, GABA, as a signaling molecule, is involved in controlling the level of other secondary messengers. The interrelationships between GABA signaling and the pathways of other regulators create a network of connections, which is the subject of current research. For this reason, the review presents the results of the latest studies on the crosstalk between GABA, plant hormones, polyamines, and other signaling molecules (including gasotransmitters, H_2_O_2_, and melatonin) and its role in both plant growth and development, as well as plant adaptation to environmental stresses. Particular attention was paid to the regulation of the enzymatic pathways involved in the modulation of endogenous GABA levels in plants. In summary, the aim of this work was to explain the action of GABA as an element of a complex signaling network involving other cellular messengers.

## 2. GABA Biosynthesis

**Figure 1:**
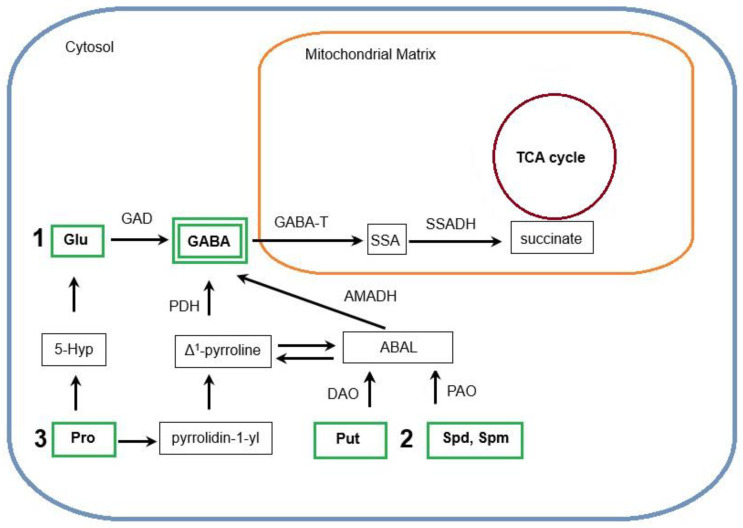
Pathways of GABA biosynthesis in plant cells. GABA can be formed (**1**) from glutamate (Glu) via the GABA shunt, including Glu decarboxylase (GAD), GABA aminotransferase (GABA-T), and succinic semialdehyde (SSA) dehydrogenase (SSADH) activities; (**2**) as a result of the oxidative degradation of polyamines: putrescine (Put), spermidine (Spd) and spermine (Spm), using diamine oxidase (DAO), polyamine oxidase (PAO), 4-aminobutyraldehyde (ABAL) dehydrogenase (AMADH), and pyrroline dehydrogenase (PDH) activities; (**3**) from proline (Pro) conversion via pyrrolidin-1-yl or 5-hydroxyproline (5-Hyp); the substrates for GABA production are marked in green.

Given that GABA has been demonstrated to accumulate in plant tissues in response to abiotic and biotic stresses [3,4,9,10,11,12,13], it is worth focusing on its biosynthesis in plants. Several modes of GABA production have been identified in plants. In the initial stages of GABA research, a metabolic pathway designated as the GABA shunt, also observed in animal models, was recognized as the primary source of its biosynthesis. This route of GABA synthesis and metabolism represents a branch of the tricarboxylic acid (TCA) cycle. The process consists of three principal reactions, which are catalyzed by the cytosolic enzyme glutamate decarboxylase (GAD) and the two mitochondrial enzymes gamma-aminobutyric acid aminotransferase (GABA-T) and succinic semialdehyde dehydrogenase (SSADH) [14], Figure 1.

Glutamic acid (Glu) is decarboxylated in the cytosol to produce GABA in a reaction catalyzed by the enzyme GAD that consumes H^+^ and releases CO_2_. Plant GAD contains a calmodulin (CaM)-binding domain that determines its in vitro activity at pH 7.0–7.5. On the contrary, at acidic pH, GAD activity is less dependent on Ca^2+^-CaM and determines an optimal pH of 5.8 [6,15]. GAD can exist in dimer or hexamer forms [8,16]. GAD activity exhibits a low level under physiological conditions, while it increases with the acidification of the cytosol, reaching an optimum pH of 5.8. A reduction in cytosolic pH below the physiological norm results in the loss of GAD’s ability to bind Ca^2+^-CaM [8,17]. Under physiological conditions, GAD is predominantly present in a dimer form, exhibiting low activity. However, alterations in pH or the attachment of Ca^2+^-CaM to the C-terminus result in a transition to the hexamer, thus increasing enzyme activity [8]. Changes in cytosolic pH and Ca^2+^ levels are common responses of plants to a variety of stresses. At normal physiological pH, an elevation of calcium ions in the cytosol causes the formation of a Ca²⁺-CaM complex, which in turn activates the GAD enzyme. This activation occurs during the mild or early stages of stress. In contrast, during acute stress and chronic stress, the pH of the cytosol decreases, leading to the activation of the GAD enzyme in a pH-dependent manner [18]. Several different GADs have been found in various plant species. For example, five GAD isoforms have been identified in *Arabidopsis thaliana* [19]. Different *GADs* are expressed in a tissue-dependent manner. In *Arabidopsis*, it has been observed that *GAD1* is expressed primarily in roots, whereas *GAD2* in all the organs [20]. Furthermore, the expression of genes encoding different GAD isoforms also depends on the stress factors that affect plants. The expression of *GAD1* and *GAD5* was shown to be upregulated in response to a multitude of stressors, whereas the levels of *GAD3* and *GAD4* are exclusively stimulated by salinity, dehydration, and UV-B [21]. In cotton, 10 genes encoding GAD isoforms have been identified. Furthermore, *GhGAD6* has been shown to be functionally responsive to cadmium stress [22]. There are four principal phylogenetic subfamilies of *GADs*. The third subfamily is the most abundant [22]. The *Arabidopsis GAD* isoforms are members of the first and third subfamilies. Four of them, *AtGAD1*, *AtGAD2*, *AtGAD3*, and *AtGAD4*, are represented in the third subfamily. In contrast, cotton *GAD* isoforms are present in the first, second, and third subfamilies. Of these, six are assigned to the third subfamily [22].

The subsequent phase of the GABA shunt occurs within the mitochondria, where GABA is transferred by a specific transporter, mitochondrial GABA permease (GABAP) [23]. In these organelles, it is converted to succinimide semialdehyde (SSA) by GABA-T and finally to succinate by SSADH [23], Figure 1. A variety of GABA-Ts have been identified in plants. In the catabolism of GABA in the mitochondrial matrix, GABA-T can use a variety of amine acceptors, including alpha-ketoglutarate, pyruvate, and glyoxalate [20,23]. Depending on substrate affinity, GABA-TK transaminases can catalyze an alpha-ketoglutarate-dependent reaction and generate SSA and glutamate, while GABA-TPs receive an amino group from pyruvate or glyoxalate to produce alanine or glycine, respectively [24]. Additionally, GABA-Ts are present not only in mitochondria but also in plastids and the cytoplasm [8]. In tomato, three *GABA-TP* genes have been suggested to be involved in catabolizing GABA to SSA: *SlGABA-T1* in mitochondria, *SlGABA-T2* in the cytosol, and *SlGABA-T3* in plastids [24]. Among them, *SlGABA-T1* was characterized by the highest expression level. Thus, the mitochondrial form of GABA-TP was suggested to be the most important in GABA metabolism at least in tomato [25]. Only loss-of-function by *SlGABA-T1* showed a 9-fold increase in GABA content in tomato fruits. In contrast, no increase in GABA accumulation was observed for mutations in the other two genes expressed in the plastids and cytosol [24]. Similarly, in *Arabidopsis thaliana*, *GABA-T* knockout mutants exhibited elevated GABA levels [8]. *GABA-T* expression, similarly to *GAD*, is modified by various stress factors, e.g., UV-B, drought, low temperature, salt, and cadmium [21,26]. The activity of GABA-T is regulated by the ratio of oxidized to reduced NAD. A decrease in the NAD^+^/NADH ratio results in the inhibition of both the activity of SSADH and succinate production. This leads to the accumulation of SSA, which in turn inhibits GABA-T activity [27].

The GABA shunt appears to be the main mechanism of the synthesis of GABA and the maintenance of its optimal concentration in plant tissues [14]. Furthermore, alternative routes for GABA production have been identified. Under stress conditions, a notable increase in GABA accumulation is associated with polyamine (PA) catabolism, Figure 1. In plants, specific oxidases including diamine oxidase (DAO) and polyamine oxidase (PAO) are responsible for PA degradation. DAOs (copper-containing amine oxidases, CuAOs) oxidize primary PAs (mainly diamine putrescine, Put) whereas FAD-dependent PAOs catalyze the oxidation of secondary and tertiary PAs (triamine spermidine, Spd; tetraamine spermine, Spm; and thermospermine, Tspm). These reactions produce 4-aminobutyraldehyde (4-aminobutanal, ABAL) [28]. ABAL cyclizes to form Δ^1^-pyrroline, both are in equilibrium and can be spontaneously interconverted. In the next step, ABAL/Δ^1^-pyrroline undergoes dehydrogenation via the action of NAD^+^-driven 4-aminobutyraldehyde dehydrogenase (AMADH) or pyrroline dehydrogenase (PDH), respectively, releasing GABA [27,29,30]. Therefore, changes in the content of endogenous PAs, especially Put, as well as in the activities of DAO, PAO, AMADH, and PDH can affect the accumulation of GABA in plant cells. As an example, GABA has been shown to act as a key player in the acclimation of *Arabidopsis* to combined high light and heat stress. The stress-induced accumulation of GABA occurred in parallel with a decrease in Put level, suggesting that PA oxidation may contribute to the generation of this molecule [31].

Additionally, GABA can be synthesized via a non-enzymatic reaction from proline (Pro) [32], Figure 1. Pro has been demonstrated to play a crucial role in the response of plants to a variety of biotic and abiotic stresses [33]. Under such circumstances, the accumulation of this compound increases markedly. Furthermore, it is important to note that stress conditions contribute to the production of an excess of reactive oxygen species (ROS), which can negatively affect plant cells. Pro has the ability to function as a scavenger of hydroxyl radicals (HO^•^). Amici et al. [34] demonstrated that a reaction between HO^•^ and Pro results in the formation of 5-hydroxyproline (5-Hyp), which subsequently contributes to Glu production. In contrast, Signorelli et al. [32] demonstrated that Pro reacts with hydroxyl groups (OH^−^), resulting in the spontaneous decarboxylation of Pro and the formation of pyrrolidin-1-yl. Pyrrolidin-1-yl can undergo conversion to Δ^1^-pyrroline, which serves as a substrate for the enzyme PDH, which in turn produces GABA. This represents an alternative, non-enzymatic pathway for the synthesis of GABA, at least under the conditions of oxidative stress [32].

## 3. Interactions between GABA and Phytohormones

Plant hormones (phytohormones) function as signaling molecules involved in the proper growth and development of plant organisms. They are known to regulate seed germination/dormancy, flowering, fruit ripening, and leaf abscission/senescence. Acting as growth stimulators or inhibitors, they control the elongation of plant organs and are responsible for the architecture of roots and shoots [35]. They participate in various cellular processes, including stomatal movements [36]. On the other hand, phytohormones play a key role in plant responses to stress factors, both biotic and abiotic. Modulations in their levels allow plants to adapt to unfavorable environmental conditions [37].

**Figure 2:**
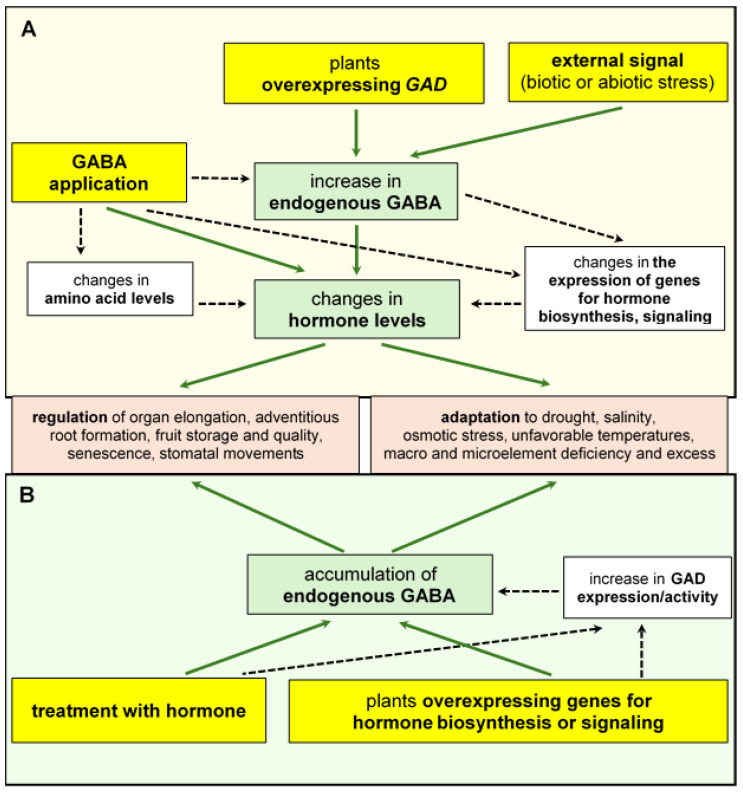
Interactions between phytohormones and GABA. GABA can act both upstream (**A**) and downstream (**B**) of hormones, regulating plant growth and development, as well as triggering stress tolerance. An increase in endogenous GABA, related to GABA application, the overexpression of *GAD* (encoding glutamate decarboxylase), or environmental stress factor, was shown to enhance the content of phytohormones, and, in this way, regulate cellular processes. On the other hand, plant treatment with phytohormones and their increased production/signaling lead to GABA accumulation and its enhanced function (the external sources of GABA and phytohormones are highlighted in yellow, and their endogenous accumulation in green; the green arrows indicate the direction of the main changes/relationships identified in the studies, while the black dashed arrows the possibility of additional relations).

More and more data indicate that GABA, like phytohormones, accumulates in plant tissues in response to different environmental signals, affecting plant growth and development. Furthermore, exogenous GABA was shown to modify the expression of the genes involved in phytohormone-responsive signal transduction and metabolism under normal and stress conditions [27]. The application of GABA may have a simultaneous effect on the accumulation of various plant hormones. A significant increase in phytohormone levels was observed in sweet orange (*Citrus sinensis*) leaves after treating plants with GABA [38]. GABA enhanced the contents of salicylic acid (SA) and its precursors: benzoic acid (BA) and cinnamic acid (CA); jasmonic acid (JA); auxins such as indole-3-acetic acid (IAA) and indole-3-propionic acid (IPA); and abscisic acid (ABA). The observed effects were at least partially related to the activated expression of genes encoding biosynthetic enzymes. The analysis of 16 selected genes involved in auxin (e.g., *CitTAA* and *CitYUC2*), salicylate (e.g., *CitPAL*, *CitCS*, and *CitICS*), and jasmonate (e.g., *CitLOX*, *CitAOS*, and *CitAOC*) synthesis confirmed their GABA-induced upregulation [38]. Moreover, the authors suggested that enhanced hormone production may also result from a GABA-promoted increase in the levels of several amino acids that function as substrates for the production of phytohormones, Figure 2.

On the other hand, the treatment of plants with hormones was found to change the accumulation of GABA in plants. Exposure to auxins, including IAA and NAA (α-naphthaleneacetic acid), enhanced endogenous GABA in excised root tips of rice [39] and root cultures of *Datura stramonium* [40] while decreasing it in *Arabidopsis* roots [41]. Similarly, ABA elevated the GABA level in wheat seedlings [42]. This suggests that both types of molecules can interact with each other, coordinating various physiological processes in plant cells and enabling plants to adapt and survive [27,43]. See Figure 2.

### 3.1. Processes of Plant Growth and Development

During plant growth and development, GABA often appears to function as a downregulator of many processes. Comparing *Arabidopsis* wild-type plants and mutants defective in GABA catabolism (*pop2*), Renault et al. [44] showed a significant increase in the expression level of the genes related to hormone metabolism in *pop2-1* treated with exogenous GABA. They confirmed the inhibitory effect of GABA accumulation on root cell growth and proposed that GABA does not work independently, but together with phytohormones to control the elongation of plant organs.

GABA was found to be a negative regulator of adventitious root (AR) development [45]. Studies using transgenic lines with the overexpression of *PagGAD2* and vigabatrin, a specific GABA-T inhibitor, revealed that in poplar (*Populus alba × P. glandulosa*), GABA induces changes in the level of phytohormones and the expression of the genes involved in their synthesis and signal transduction during AR formation [45]. High levels of endogenous GABA resulted in increased IAA accumulation and the transcriptional upregulation of genes involved in the auxin pathway (including *YUC*, *GH3*, *Aux/IAA*, *ARF*, and *ABP*). Similarly, the ABA content was enhanced and the expression of ABA-dependent genes (including *ZEP*, *PYR*, and *PP2C*) was modified as a consequence of elevated GABA levels. In contrast, a decrease in ethylene concentration, not related to the downregulation of *ERF* genes, was shown [45]. Similarly, in poplar tissue cultures, when GABA shunt activity was promoted, AR growth was stunted [46]. Under such conditions, the levels of IAA, GA_3_ (gibberellin 3), ABA, and ethylene did not change significantly in the roots compared to the control. However, both endogenous auxins and gibberellins (GAs) increased, whereas ethylene decreased in stems. An analysis of the expression patterns of the genes involved in phytohormone homeostasis in poplar roots showed that increased GABA shunt activity causes a significant difference in the transcription of the genes related to the production and signaling of IAA, ABA, ethylene, and GA [46]. These results indicated that the close relationship between GABA and hormonal pathways is responsible for the delay in AR development observed in poplar.

In addition, Li et al. [47] confirmed that the GABA-induced inhibition of AR formation is associated with the auxin response in apple (*Malus xiaojinensis*). Treatment with IBA (indole-3-butyric acid) stimulated the accumulation of GABA and the activity and gene expression of GAD in adult seedlings, but not in juvenile ones. Furthermore, *MxPIN* gene transcription was enhanced in response to exogenous IBA. However, this auxin-dependent upregulation was diminished by GABA application, suggesting that GABA delays AR development by reducing polar auxin transport [47]. Therefore, in general, GABA-altered auxin homeostasis and distribution appear to be one of the factors responsible for AR occurrence during the initial phases of the rooting process [48].

GAs interrupt seed dormancy and promote the germination process [49]. For this reason, their effects on GABA accumulation were studied in germinated brown rice (GBR), used as a high-GABA functional food [50]. The seeds of two rice genotypes (indica and japonica) were treated with GA_3_ or Glu, a substrate for GABA production. GABA levels were found to increase with the addition of GA_3_ to the soaking water. However, higher GABA accumulation was observed in the indica cultivar. Moreover, GA_3_ had a similar effect as Glu; both substances shortened the optimal germination time by 6 h. The addition of GA (or Glu) to seeds has been proposed to be a way to achieve the lower endogenous GABA accumulation in *Oryza sativa japonica* [50].

Interactions between GABA metabolism and GA pathways were confirmed in poplar *gai* and *rgl1* dwarfs overexpressing mutated *Arabidopsis* GAI or RGL proteins, respectively. Both proteins belong to DELLA repressors involved in GA signaling. Significant changes in the levels of metabolites, including GABA, were found in transgenic plants with impaired GA response compared to wild-type plants. The *gai* and *rgl1* mutants showed more than three-fold GABA accumulation in roots and its reduction in leaves [51]. Moreover, in the highly drought-tolerant fungus *Aurcularia fbrillifera*, the genes related to both GA and GABA synthesis (encoding ent-kaurene oxidase and glutamate decarboxylase, respectively) were upregulated during rehydration, indicating their essential role in breaking dormancy and the recovery of growth after rehydration [52].

The possible role of GABA and its interrelation with GAs during the initiation of the parthenocarpic fruit-set was studied in grapevines (*Vitis* spp.) [53]. Before full bloom, endogenous GABA was maintained at a constant level by the upregulation of *VvGAD1* and downregulation of *VvGABA-T2*. The treatment of plants with GAs resulted in an earlier increase in *VvGAD1* expression and, consequently, GABA overproduction during full blooming [53]. The authors suggested that the GA-induced GABA imbalance may be responsible for the inhibition of pollen tube growth and, finally, the disturbance of crosstalk between pistil and pollen.

The accumulation of GABA in plant tissues is often accompanied by the enhanced biosynthesis of ethylene [54]. For this reason, much attention has been paid to the connections between the functions of these two molecules. GABA was shown to significantly increase ethylene production in excised sunflower tissues [55]. This effect was not the result of GABA acting as a potential ethylene precursor. However, a GABA-induced increase in both ACC (1-aminocyclopropane-1-carboxylate) synthase (ACS) and ACC oxidase (ACO) gene expression was observed. The authors suggested that GABA promotes ethylene production by affecting the level of ACC, the main substrate for its synthesis in plants [55]. Similarly, high concentrations of GABA stimulated the transcription of the *ACS* gene and the production of ethylene in the stems of *Stellaria longipes*. GABA-induced ethylene accumulation was responsible for the observed inhibition of stem elongation [56]. On the other hand, reduced ethylene levels have also been observed in GABA-treated plant organs, for example, in detached soybean leaves [57] and in apple fruits [58,59]. In apple, a decrease in ethylene production was related to the downregulation of the genes involved in ethylene synthesis (*MdACS* and *MdACO*) and signaling (*MdERF*), suggesting possible crosstalk between the GABA and ethylene pathways during fruit storage to maintain their quality [58,59]. This indicates that GABA may delay some processes, e.g., senescence, by inhibiting ethylene synthesis in tissues.

Both ethylene and ABA participate in the GABA-dependent regulation of 14-3-3 gene (*Grf*) expression in *Arabidopsis thaliana* [60]. GABA was shown to reduce the total level of cellular 14-3-3 proteins and repress the transcription of most *Grf* genes at high calcium. This effect was abolished in the *abi1-1* and *abi2-1* mutants, defective in ABA signaling, as well as in the ethylene-insensitive *etr1* mutant. Moreover, providing plants with a substrate for ethylene synthesis (ACC) also led to the loss of the GABA-induced downregulation of *Grf* expression. This suggests that both excessive and weakened ethylene signaling result in a lack of response to GABA. The authors concluded that ABA- and ethylene-dependent signal transduction plays a key role in the GABA pathway and acts downstream of GABA in the regulation of 14-3-3 gene expression [60].

Similar functions of GABA and SA in delaying the senescence of cut flowers were shown in tuberose [61]. The application of each of both molecules had a positive effect on the vase life and improved the post-harvest quality of the flowers, including water absorption, fresh weight, and chlorophyll and protein content. Moreover, the activities of the antioxidant enzymes were enhanced, indicating that GABA and SA can postpone senescence by activating the antioxidant system [61]. However, the relationship between SA and GABA signaling in the regulation of this process has not yet been demonstrated.

Previously, jasmonates were shown to induce tendril coiling in *Bryonia dioica* [62], but not in other plants. Therefore, Malabarba et al. [63] investigated the role of jasmonates, including JA, jasmonoyl-isoleucine (JA-Ile), and its precursor 12-oxophytodienoic acid (OPDA), in this process in grapevine and compared it with the function of GABA. The levels of OPDA, JA, and JA-Ile were visibly higher in non-coiled than in coiled tendrils. Similarly, non-coiled tendrils showed elevated ABA content. In contrast, GABA accumulated at significantly high levels in both coiled and non-coiled tendrils, suggesting that this molecule is involved in grapevine tendril growth/development. When non-coiled tendrils were treated with exogenous JA or GABA, the induction of the coiling process (without touching) was observed. However, simultaneous exposure to both molecules had no additive or synergistic effect [63]. Thus, it was suggested that JA and GABA act independently in promoting tendril coiling in grapevine.

It has been proposed that GABA acts as a universal modulator of stomatal movements in dicotyledonous and monocotyledonous plants [64]. *Arabidopsis gad1/2* mutants, with reduced GABA content, showed increased stomatal aperture and conductance, as well as impaired stomatal closure [65]. Exogenous GABA and its analog muscimol inhibited light-dependent stomatal opening and dark-dependent stomatal closure. The effect of both compounds was opposite to that of the other known regulators, including phytohormones. They were able to suppress the stomatal closure induced by ABA and stomatal opening activated by coronatine (which mimics JA). In contrast, GABA and muscimol had no effect on the stomatal pores that remained closed at high ABA concentrations [64]. Moreover, it was suggested that the interaction between GABA and ALMT (aluminum-activated malate transporter) proteins, putative GABA receptors located in the vacuolar and plasma membranes, is involved in the regulation of stomatal movement [65]. In *Arabidopsis* double mutants *gad2-1/almt9-1* and *gad2-1/almt9-2*, characterized by lowered GABA content in leaves, ABA was shown to induce stomatal closure to levels observed in wild-type plants [64]. Therefore, the crosstalk between GABA, ALMT9, and ABA plays an important role in this process. On the other hand, the expression of *ALMT1*, present mainly in *Arabidopsis* roots, was induced by IAA and ABA [66], confirming the possibility of phytohormone interaction with ALMT proteins.

### 3.2. Plant Responses to Abiotic and Biotic Stress

Since both GABA- and phytohormone-related pathways can be activated under unfavorable conditions, Zheng et al. [67] identified three gene families (*GAD*, *GAB-T*, and *SSADH*) of the GABA branch in four cotton varieties (including *Gossypium hirsutum*, *G. barbadense*, *G. arboreum*, and *G. raimondii*) and analyzed them with respect to the plant reaction to abiotic stress. The analysis of promoter sequences indicated that all genes contain hormone- and environment-responsive regulatory elements. Among them, the most abundant elements were those related to auxins, ABA, GAs, and SA, suggesting that GABA metabolism may be controlled by phytohormones [67].

#### 3.2.1. Drought

Using six geographical and climatological *Pinus radiata* breeds with different drought tolerance, it was found that GABA and phytohormones synergistically accumulated in plant needles during drought hardening [68]. Although differences were found between breeds, a marked increase in the levels of IAA, ABA, SA, JA, and zeatin riboside (ZR) was observed. This indicates a possible protective role for GABA and hormones in alleviating and delaying the harmful effects of drought [68].

The contribution of GABA-, ABA-, and SA-dependent metabolic changes in drought tolerance was determined in creeping bentgrass (*Agrostis stolonifera*) [69]. The application of each of the regulatory molecules mitigated stress-related damage by maintaining membrane stability and water status in leaves. On the one hand, GABA, ABA, and SA affected common metabolic pathways, but on the other hand, they caused different changes in metabolic profiles under stress conditions. GABA was found to stimulate the biosynthesis of amino acids and organic acids. ABA mainly enhanced the level of organic acids for the TCA cycle, whereas SA significantly increased the accumulation of both amino acids and carbohydrates. The authors concluded that the drought resistance of bentgrass could be attributed to the activation of secondary metabolism involved in the defense response (induced by GABA), increased respiratory metabolism (associated with ABA and SA), and osmotic adjustment (related to SA and GABA) [69]. Moreover, drought-related metabolic changes were analyzed in transgenic creeping bentgrass with the overexpression of *ipt* (encoding isopentenyl transferase) and elevated cytokinin (CK) level [70]. Plant transformation improved drought tolerance, which was correlated with the accumulation of several amino acids, carbohydrates, and organic acids. Water scarcity resulted in increased GABA production in the leaves of *ipt* plants, in contrast to null transformants, which showed a significant decrease in GABA content. The authors suggested that CK signaling may, at least in part, alter the GABA pathway through changes in the content of pyroglutamic acid and Pro, GABA precursors [70].

It was shown that exogenous GABA promotes the accumulation of GABA in the leaves of apple seedlings subjected to drought conditions [71]. As a consequence, a significant reduction in stomatal conductance and transpiration rate, as well as an increase in stomatal closure and photosynthesis rate were observed. GABA upregulated the expression of the genes involved in ABA signaling, including *ABI1*, *ABI2*, *ABF3*, and *OST1*. It has been suggested that the GABA-related regulation of endogenous ABA levels may be important to improve drought tolerance in apples [71].

In *Arabidopsis*, drought stress tolerance can be enhanced by the overexpression of *BRL3* encoding a vascular tissue-specific brassinosteroid (BR) receptor [72]. *BRL3* overexpression led to changes in the profiles of metabolites important for osmoprotective processes, especially sugars and amino acids. Under drought stress, the shoots of the *BRL3ox* mutants showed an increased content of GABA, Pro, and Tyr. This suggests a positive correlation between GABA and BR signaling in plant adaptation to water deficit [72].

#### 3.2.2. Salinity, Osmotic Stress, and Unfavorable Temperatures

The close relationship between GABA and various phytohormone signaling pathways has been proposed to play a significant role in plant salinity tolerance [73]. Salt stress caused visible changes in the phytohormone profile in the leaves of *Cassia italica* seedlings [74]. Under salinity conditions, the levels of growth-related hormones, including auxins (IAA and IBA) and gibberellins (GA_1_ and GA_4_), were reduced, while the concentrations of stress-related hormones such as ABA and JA were increased. The priming of seeds in GABA solution resulted in an enhancement of auxin and GA content and a decrease in ABA and JA amount. Therefore, it was suggested that GABA helps maintain hormonal balance in salt-stressed plants [74].

Exogenous GABA was found to regulate the expression of *ACO* genes and ethylene synthesis in the NaCl-treated roots of *Caragana intermedia* [2]. Moreover, in this plant, GABA also altered the expression of some regulatory genes with a high homology to *GPCR* (encoding ABA receptor) and *ABA2* (encoding short-chain dehydrogenase/reductase) in response to NaCl stress [2]. Ethylene levels were modulated by GABA in a concentration- and time-dependent manner in poplar seedlings subjected to salinity [75]. GABA affected the expression of 6 *ACO* genes, 4 *ACS* genes, 2 *ETR* genes encoding ethylene receptor, and about 40 genes encoding ethylene-responsive transcription factors. Since the transcription of most *ERF*s was positively correlated with ethylene accumulation, the authors proposed that GABA can regulate downstream ethylene signaling by controlling its endogenous content in plant tissues exposed to salt stress [75]. Parallel with changes in ethylene synthesis, GABA influenced ABA accumulation and also regulated the expression of three genes (*ABAH* and *ABAG*) involved in ABA metabolism and four genes (*PYL*) involved in ABA perception in salt-stressed poplar leaves and roots. This indicates that GABA may affect ABA signaling by controlling ABA production and regulating ABA receptor gene expression under salt stress [75].

To alleviate the adverse effects of salinity on medicinal pumpkin (*Cucurbita pepo*), seedlings were pretreated with epibrassinolide (EBL), one of the BRs that naturally occurs in plants [76]. Under salt stress, a significant increase in GABA content in leaves and roots was demonstrated. As a result of plant pretreatment with EBL, GABA levels were further enhanced, in parallel with reduced lipid peroxidation and the activation of antioxidant enzymes. This emphasizes that EBL can protect plants against the harmful effects of salt, among others, by controlling the accumulation of GABA [76]. The possible role of brassinolide (BL), GABA, and SA in improving the growth and development of plants growing under salinity conditions has also been investigated in grapevine [77]. The plants were treated with regulatory substances by foliar spraying. All the regulators were found to alleviate unfavorable salt-induced changes in growth parameters, photosynthetic properties, and berry quality. At the same time, it was observed that individual compounds affected other aspects of fruit ripening. Therefore, it seems important to investigate the relationship between the GABA, BR, and SA pathways in the future. Zao et al. [77] concluded that the obtained results are the basis for the use of exogenously applied regulators in grapevine cultivation to improve plant parameters.

The protective role of GABA and SA in the adaptation to salinity and high temperature was investigated in *Origanum vulgare* [78]. The analyses of various physiological, biochemical, and phytochemical parameters (including photosynthetic pigments, oxidative stress indicators, antioxidant response, and essential oil composition) showed that although both compounds have a positive effect on stress tolerance, GABA seems to be more effective under salt stress whereas SA has a greater protective effect on plants growing under heat stress. The possible relationship between SA and GABA metabolism was demonstrated in pepper (*Capsicum annuum* L.) plants exposed to salinity and osmotic stress [79]. SA was shown to not promote the production of the glutamate pool during both individual and combined stress conditions. On the other hand, it increased GAD activity, involved in the conversion of glutamate to GABA, under PEG-induced osmotic stress. The determination of the GABA level indicated that exogenous SA had no beneficial effect on GABA accumulation in pepper leaves [79].

To understand the mechanisms responsible for the chilling injury (seed browning) of pepper fruit and to develop strategies to maintain their post-harvest freshness during cold storage, the endogenous level and related gene expression of different hormones, including IAA, ABA, GA, JA, JA-Ile, and SA, and their relationship with amino acid content were determined [80]. Two genotypes of *C. annuum* seeds with different sensitivity to low temperatures were compared. It was found that changes in auxin levels were correlated with susceptibility to cold-induced injury. The chilling-sensitive genotype showed a high IAA content during storage, while no changes in the IAA content were observed in the less sensitive genotype. In addition, an increase in endogenous GABA was demonstrated in both pepper cultivars during cold storage. In the less sensitive genotype, the level of chilling injury was related to the GABA content and the auxin response factor *CaARF19*. In the more sensitive one, there was a strong correlation between seed browning, GABA level, auxin response factor *CaARF6*, and *CaTIR1* encoding the auxin receptor [80]. Therefore, IAA and possibly its interaction with other factors, including GABA, appear to be involved in the cold-induced injury of pepper fruit.

#### 3.2.3. Deficiency and Excess of Macro and Microelements

GABA can alleviate the adverse effects of mineral deficiencies in plants. This molecule has been shown to improve cucumber tolerance to iron deficit in an auxin-dependent manner [81]. GABA-treated seedlings, growing under Fe shortage, showed a significant increase in IAA content at the shoot apex. This increase was correlated with the upregulation of the genes involved in auxin biosynthesis (*YUC4*), signaling (*IAA1*), and transport (*PIN1*). The relationship between auxin and GABA pathways was confirmed using an auxin transport inhibitor, NPA (1-naphthylphthalamic acid). Its application abolished the stimulatory effects of GABA on various physiological responses, including chlorophyll content, root ferric-chelate reductase (FCR) activity, and the expression of the genes involved in iron uptake and reduction, *IRT1* and *FRO2*, respectively [81]. The authors concluded that GABA promotes IAA production in shoots and its polar transport, which, consequently, induces Fe uptake and reduction in roots.

Similarly, crosstalk between GABA and auxins was demonstrated in apple seedlings subjected to phosphorus deficiency [82]. Exogenous GABA alleviated the disadvantageous effects of phosphorus starvation by improving plant growth, root development, photosynthetic efficiency, and P uptake. Moreover, this molecule increased the IAA content in the roots. Transgenic apple roots, with *MdGAD1* overexpression and enhanced endogenous GABA, exposed to low P, showed both a higher level of IAA and the upregulation of genes related to auxin pathways, including *IAA1*, *YUCCA4*, *YUCCA6*, *AUX1*, and *PIN1* [82]. The results indicated that under P deficiency conditions, GABA has a beneficial effect on root growth and root tip cell development, and acts through auxin signaling.

Transgenic tobacco plants overexpressing the *ipt* gene showed increased CK levels in tissues and improved tolerance to zinc-induced stress [83]. The analysis of the content of selected amino acids involved in plant senescence and adaptation confirmed that enhanced CK production resulted in changes in the amino acid profile, including GABA. GABA levels decreased in transgenic plants compared to wild-type tobacco. However, under zinc stress, endogenous GABA remained at a constant level in mutants, while in untransformed plants, it was significantly reduced [83]. This suggests that the increase in Zn tolerance is at least partially related to the accumulation of CK and GABA.

Strigolactones (SLs) are another group of plant hormones derived from carotenoids. The relations of SL with GABA, JA, and nitrogen deficit (ND) were investigated in the microalga *Monoraphidium* [84]. It was reported that under nitrogen deficiency, the JA content increased whereas the GABA level decreased regardless of supplementation with SL (GR24, an SL analog). On the other hand, GABA accumulation and lipid content were significantly stimulated in ND- and SL-exposed cells treated with JA, suggesting that under such conditions, JA acts via GABA signaling [84]. The authors proposed that the combination of SL with ND could promote cell growth and lipid biosynthesis in *Monoraphidium* through endogenous JA.

#### 3.2.4. Biotic Stress

Studies using *A. thaliana gad1/2* double mutants with reduced GABA levels and *gad1/2* × *pop2-5* triple mutants with enriched endogenous GABA showed that GABA accumulates in leaves after insect attack [85]. Such effect was achieved by both insect feeding-like wounding and the cotton leafworm (*Spodoptera littoralis*). An increase in JA and JA-Ile content was also observed as a result of larval feeding. However, no significant differences were found between wild-type and mutant plants. To evaluate the relationship between GABA- and JA-related defense, wild-type plants were treated with coronalon, a synthetic jasmonate that mimics JA-Ile, and an *Arabidopsis jar1* mutant, defective in JA-Ile synthesis, was used. Coronalon did not induce any changes in GABA accumulation. Moreover, the *jar1* mutant showed significantly higher GABA levels than WT plants. Therefore, it could be concluded that GABA had no effect on jasmonate accumulation and, similarly, GABA production was not dependent on jasmonate [85]. In addition, the role of jasmonates in GABA generation was analyzed in *Arabidopsis pop2-5* single mutants. The content of JA and JA-Ile was visibly enhanced after feeding with *S. littoralis* and wounding, but differences between WT and mutants were not observed [10]. The obtained results confirm that jasmonate-independent GABA accumulation occurs in *Arabidopsis* as a defense reaction against insect herbivores [85]. However, the authors did not exclude the possibility that the octadecane pathway independent of JA/JA-Ile may be involved in this process.

Similar observations were made by Mirabella et al. [86] in the seedlings of the *A. thaliana her1* mutant. This mutant showed an altered response to *E-2*-hexenal, a member of C6-volatiles, a group of six-carbon alcohols, aldehydes, and esters produced by plants when attacked by pathogens or herbivores. In contrast to WT plants, *E-2*-hexenal did not inhibit root elongation in *her1* plants. Because *her1* was found to encode a GABA-T, the mutant plants had a higher GABA content in their tissues. The analysis of the root response of different *A. thaliana* mutants, defective in JA, SA, and ethylene signaling or biosynthesis, confirmed that *E-2*-hexenal-induced root growth inhibition was not related to the pathways of these phytohormones. It can be concluded that GABA enhances tolerance to *E-2*-hexenal through JA-, SA-, and ethylene-independent signaling [86].

## 4. Polyamines and Their Relation to the Action of GABA

PAs are phytohormone-like plant growth regulators. They were found to play an important role in both controlling plant growth and development and triggering stress responses [87]. It is well known that PA-dependent signaling has a beneficial effect in improving crop productivity and resistance to adverse environmental changes [88]. Similarly to phytohormones, PAs interact with other secondary messengers that function in plant cells, regulating physiological processes [87].

PAs and GABA can act in parallel to produce similar effects in plants. PAs together with GABA confer plant tolerance to low-temperature stress, which is associated with the cold-induced reprogramming of the cellular metabolic network. As a consequence, antioxidant defense is activated, mitigating ROS-dependent damage [89]. Moreover, both GABA and PA metabolism can be regulated by NO, leading to increased cold tolerance [90]. Changes in the levels of PAs and soluble amino acids, including GABA, are also essential for drought resistance. A comparison of six *Pinia radiata* breeds, varied in drought tolerance, indicated significant differences in the concentration of several metabolites after hardening [68]. Although GABA, Put, and Spm accumulated at high levels in all the breeds after the second drought cycle, *P. radiata* var. *radiata* × *P. radiata* var. *cedrosensis* showed the largest amounts of these compounds. Therefore, drought hardening appears to be an intraspecifically controlled process, dependent on breed-related metabolic changes [68].

Both PAs and GABA have been shown to act as anti-aging agents. They delay senescence and extend the vase life of cut flowers. Pre-harvest spraying with Spm or GABA helps maintain the post-harvest quality of gerbera flowers (commercial cultivar “Stanza”) by increasing the antioxidant response and improving growth parameters [91]. Moreover, treatment with each of these two substances significantly prevented protein degradation and cell damage induced in flowers during cold storage. Compared to Spm, GABA mediated chilling tolerance by additionally increasing Pro and total protein synthesis [92]. Kim et al. [93] reported the involvement of PA- and GABA-dependent signaling pathways in hypersensitive cell death induced by AvrBsT, a type III effector protein of *Xanthomonas campestris* pv *vesicatoria* (*Xcv*), in pepper plants. Arginine decarboxylase (CaADC1), responsible for PA synthesis, has been identified as a protein interacting with AvrBsT and a key defense regulator that modulates PA and GABA metabolism. The transient expression of *CaADC1* in *Nicotiana benthamiana* leaves triggered PA production, NO and H_2_O_2_ bursts, and cell death. On the other hand, the leaves of *CaADC1*-silenced pepper plants showed decreased levels of PAs and GABA during infection. The treatment of such leaves with GABA significantly reduced avirulent *Xcv* growth [93].

### 4.1. Relationship between GABA Action and PA Degradation

**Figure 3:**
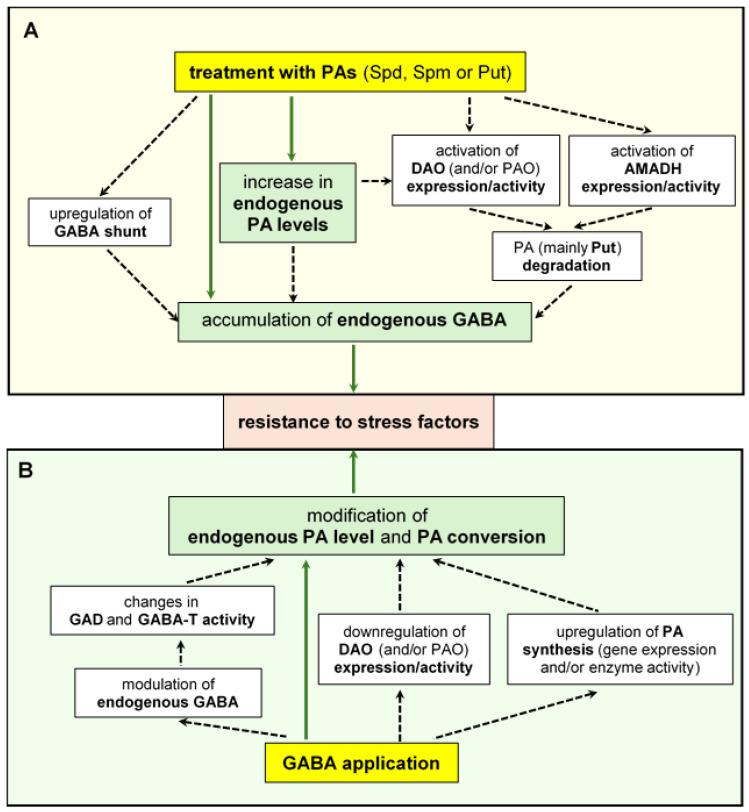
Relations between polyamines and GABA. Both polyamines (PAs) and GABA affect each other’s signaling pathways. PAs control GABA production (**A**), while GABA regulates PA level (**B**). Putrescine (Put), spermidine (Spd), and spermine (Spm) upregulate the activity and gene expression of diamine oxidase (DAO), polyamine oxidase (PAO), and 4-aminobutyraldehyde dehydrogenase (AMADH), leading to PA degradation and GABA accumulation. Moreover, PAs were found to activate the GABA shunt. On the other hand, GABA affects PA biosynthesis and degradation, and changes GABA shunt activities (both GAD and GABA-T), modulating endogenous PA content in cells (the external sources of GABA and PAs are highlighted in yellow, and their endogenous accumulation in green; the green arrows indicate the direction of the main changes/relationships identified in the studies, while the black dashed arrows the possibility of additional relations).

Both PA pathways and GABA signaling may be interconnected [94]. Since GABA can be generated in plants as a product of PA catabolism, it is not surprising that both groups of compounds can act in an interdependent manner, Figure 3.

Direct evidence that DAO activity and Put degradation are responsible for the production of GABA is provided by studies using the enzyme inhibitor, aminoguanidine (AG). In some plants, a decrease in DAO activity was found to be correlated with increased Put content and reduced GABA level [30]. On the basis of the inhibition of DAO activity, the contribution of the PA catabolic pathway in the generation of GABA was determined in several plants under various conditions. In the roots of salt-stressed soybean seedlings, 39% of the accumulated GABA was related to DAO-dependent PA catabolism [95]. Moreover, in the cotyledons and embryos of germinated soybeans exposed to hypoxia-NaCl stress, it was shown that about 33% and 36% of the GABA formation, respectively, was associated with PA degradation [96]. In fava bean, both DAO activity and GABA level were significantly enhanced during the germination process. AG treatment abolished enzyme function and decreased GABA content in seeds germinating under control conditions [97], hypoxia [98], and combined hypoxia-NaCl stress [96,99,100]. The authors concluded that in *Vicia faba*, at least about 30% of the GABA production is provided via the PA catabolic pathway during germination. A significant increase in the concentration of endogenous GABA and PAs (Put and Spm) as well as DAO activity was demonstrated in the leaves of tea plants subjected to anoxia [101]. Using AG, it was shown that about 25% of the generated GABA originates from PA catabolism. Similarly, in the roots of lupine seedlings growing under high salinity, the AG-induced inhibition of DAO activity was related to enhanced Put accumulation and decreased GABA content [102]. It has been suggested that the observed Put degradation, responsible for more than 20% of the GABA production, may be a short-term response to high NaCl concentrations.

Put-derived GABA signaling appears to play a significant role in the dark-induced senescence process of barley leaves [103]. Initially, tissues incubated in the dark showed enriched levels of free Spd, Spm, and Put. This was accompanied by an increase in the activity of both DAO and PAO, and the generation of Put conjugates. When AG was used to inhibit DAO activity, an intensified effect of senescence on photosynthetic parameters was observed. The application of AG together with GABA resulted in the restoration of the measured parameters to the control values. On the other hand, the use of guazatine, a specific PAO inhibitor, had the opposite effect as compared to that caused by AG. Moreover, during senescence, the expression level of *GAD* was downregulated, suppressing the GABA shunt pathway. The obtained results indicated that Put may serve as the main source of GABA in senescing barley leaves [103]. It is believed that PAs are involved in fruit setting and early development, as well as in fruit ripening and the acquisition of quality characteristics. A decrease in the content of PAs correlated with their increased catabolism was observed during the ripening of both climacteric and non-climacteric fruits [104]. During the ripening of Trincadeira grapes, the levels of some amino acids, including Pro and GABA, were enhanced [105]. The expression of four genes encoding amine oxidases was also shown to be upregulated, suggesting that the oxidative degradation of PAs and possibly PA-derived GABA play an essential role in this process.

PAs were found to regulate the establishment of arbuscular mycorrhizal (AM) symbiosis [106]. The interactions between PA, DAO, and GABA were analyzed in the roots of AM maize plants exposed to a water deficit [107]. Under water stress, the level of Put decreased significantly in AM roots compared to well-watered plants, whereas the Spm and Spd contents remained unchanged. Parallelly, DAO activity and GABA accumulation were enhanced in AM maize roots. Moreover, as a result of the water shortage, an improvement in nitrogen metabolism as well as an increase in GABA-T activity and malic acid content occurred. The authors suggested that at least two different pathways, including Put catabolism-dependent GABA production and GABA conversion-related malate formation, are important for the AM maize response to water deficiency [107].

PAs and their catabolism play a significant role in plant resistance to pathogen infection [108]. Furthermore, the interconnection between the GABA and PA metabolic pathways is believed to control ROS production and signaling during plant–pathogen interactions [109]. The analysis of two genotypes, drought-sensitive Chardonnay (CHR) and drought-tolerant Meski (MSK), indicated that the oxidative degradation of PAs is involved not only in the tolerance of grapevine to water stress, but also in the immune response to *Botrytis cinerea* [110]. An increase in drought resistance (in the MSK genotype) was correlated with changes in PA levels, as well as the upregulated activity and gene expression of PA-catabolised enzymes. At the same time, an accumulation of PA-related amino acids, among them Arg, Glu, Pro, and GABA, was observed. Water deficiency was shown to enhance susceptibility to pathogen attack; however, the more drought-tolerant variety was also less susceptible to infection. Using DAO and PAO inhibitors, AG and HEH (β-hydroxyethyl-hydrazine), respectively, it was demonstrated that grapevine defense reactions to both biotic and abiotic stresses involve PA oxidation, highlighting possible crosstalk between drought resistance and immune response signaling [110].

Although many studies emphasize the correlation between DAO activity and PA-derived GABA synthesis, AMADH, as the rate-limiting enzyme of GABA production, which catalyzes the final step of this pathway, has been proposed to be more suitable for quantifying the contribution of PA oxidation to the generation of the GABA pool [111]. Using EDC (1-(3-Dimethylaminopropyl)-3-ethylcarbodiimide) as a proper AMADH inhibitor, it was shown that the decrease in GABA level was associated with lowered enzyme activity in soybean sprouts under different NaCl/CaCl_2_ conditions. More than 40% of the GABA accumulated after NaCl treatment resulted from PA catabolism. The addition of CaCl_2_ decreased this proportion [111]. Two *Arabidopsis* AMADH homologs, *At*ALDH10A8 and *At*ALDH10A9, were expressed in *E. coli* and characterized as recombinant proteins [112]. They were found to be differently localized within transformed *Arabidopsis* protoplasts (in peroxisomes and plastids) and catalyze the conversion of both ABAL and APAL (3-aminopropanal), producing β-Ala or GABA, respectively. Under salt stress, *Arabidopsis aldh10a8* and *aldh10a9* knockout mutants showed reduced GABA levels compared to WT plants. Therefore, both AMADH isoforms, acting via Put-derived GABA signaling, have been proposed to be involved in salt tolerance [112]. Among the five DAO and eight AMADH isoforms identified in tea (*Camellia sinensis*) plants, peroxisomal CsCuAO1 and CsCuAO3, as well as plastid CsAMADH1, have been shown to be necessary for the GABA formation associated with Put oxidation [113]. In in vitro experiments, GABA accumulation was observed after Put supply due to the enzymatic activity of the recombinant proteins when both CsCuAO1 together with CsAMADH1 or CsCuAO3 together with CsAMADH1 were introduced. Moreover, the simultaneous addition of the three enzymes had the greatest stimulatory effect on GABA production, which was also confirmed by a coexpression analysis in *Nicotiana benthamiana* [113]. Subsequent studies demonstrated an important role for CsCuAO1 and CsAMADH1, related to GABA and Put metabolism, in tea drought tolerance [114]. Transgenic *Arabidopsis* lines overexpressing *CsAMADH1* or *CsCuAO1* were more resistant, while *CsAMADH1* and *CsCuAO1* knockdown plants were more sensitive to water deficiency. Put application resulted in an enhanced accumulation of GABA in overexpressing plants. Moreover, *CsAMADH1*-*CsCuAO1* coexpression caused more significant GABA production in leaves after Put treatment. GABA improved stress tolerance by enhancing chlorophyll content and antioxidant response, as well as regulating stomatal apertures. On the other hand, the GABA content decreased significantly in *CsAMADH1*- and *CsCuAO1*-silenced Arabidopsis, confirming the connection between DAO, AMADH, GABA, and drought resistance [114].

The relationship between DAO, AMADH, and GABA was also analyzed in barley seedlings exposed to UV-B radiation [115]. The application of EDC or AG, inhibiting PA degradation, significantly increased the endogenous Spd level, reducing the accumulation of phenolic acids and intensifying UV-B-induced oxidative damage. Both inhibitors diminished plant growth, suggesting that the Spd degradation product may be essential in the adaptation of barley to UV-B stress. The treatment of plants with GABA had a beneficial effect on seedling weight and length and on phenolic accumulation. Furthermore, the addition of GABA together with the AMADH inhibitor alleviated the effects caused by EDC. The results suggest that Spd catabolism and GABA formation are required to activate the biosynthesis of phenolic compounds and antioxidant defense during UV-B stress [115]. Similarly, AMADH activity was related to GABA content in pea seedlings during wound healing after mechanical injury [116]. The increase in enzyme activity was accompanied by GABA accumulation and spatially associated with intense lignification. At the same time, an enhancement in DAO and PAO activities, as well as the elevated levels of Put and Spd were observed. The results suggest that AMADH, PA oxidation, and GABA formation are involved in pea adaptation to mechanical stress [116].

On the other hand, both DAO and PAO activities were stimulated in black rice during seed germination at low temperatures. Furthermore, increased GABA production occurred. However, under the same conditions, AMADH activity decreased significantly, showing the opposite effect [117]. Since GAD activity was also upregulated, the authors concluded that the GABA shunt rather than the PA oxidative pathway was responsible for the enhanced GABA production under chilling.

### 4.2. Effect of Exogenous PAs on GABA Pathways

Exogenously applied PAs promote the accumulation of GABA and regulate its biosynthetic pathways in plant tissues, Figure 3. Spd-treated *Limonium tataricum* plants showed increased levels of Put and GABA in the roots. Experiments using labeled [1,4-^14^C]-Spd and [1,4-^14^C]-Put, as well as AG, confirmed the conversion of Spd to Put and, consequently, to GABA through DAO activity [118]. Similarly, the application of Spd enhanced the endogenous content of PAs, including Spd, Spm, and Put, and increased the accumulation of GABA and Glu in white clover leaves under heat stress [119]. Elevated GABA production resulted from both the induced PA degradation pathway (PAO and DAO activities) and the upregulated GABA shunt (GAD and GABA-T activities). Spd pretreatment improved the antioxidant response, increased chlorophyll biosynthesis and content, and promoted the expression of several *HSP70* and *HSP80* genes encoding heat shock proteins, thus triggering stress tolerance [119].

A beneficial effect of Spm application on PA, GABA, and nitrogen metabolism was demonstrated in creeping bentgrass exposed to drought [120]. Under stress conditions, exogenous Spm enhanced the accumulation of endogenous Put, Spd, and Spm and stimulated the activity of the enzymes producing and metabolizing PA, including DAO. During drought, a Spm-induced increase in both GABA and Glu levels, associated with upregulated GAD and GABA-T activity, was observed. The results indicated that Spm could promote GABA accumulation by activating both the GABA shunt and the PA degradation pathway. Moreover, Spm pretreatment ameliorated negative stress-related effects on NO_2_^-^ level, and the activities of nitrate reductase (NR), glutamine synthetase (GS), and glutamate synthase (GOGAT), suggesting that interactions between PAs and other metabolic pathways determine drought resistance [120]. When creeping bentgrass was subjected to salt stress, Spm application enabled plants to adapt to stressful conditions by inducing significant changes in metabolite profiles and reducing N^+^ accumulation in leaves [121]. Exogenous Spm increased endogenous PAs (Spm and Spd), amino acids (including GABA, Glu, and Ala), sugars, and other metabolites involved in osmotic adjustment, antioxidant response, and energy transformation. It was concluded that the Spm-related activation of the GABA shunt in association with a more efficient TCA cycle confers salt stress tolerance [121]. Similar advantages of using Spm were found in creeping bentgrass growing under water and heat stress [122]. The global reprogramming of metabolites resulted in improved plant growth, water relations, and photosynthesis, as well as reduced oxidative damage. Metabolomic changes concerned, among others, the compounds involved in the GABA shunt and the TCA cycle. In pumpkin seedlings, Spm pretreatment also mitigated the adverse effects induced by salt [123]. Under salinity conditions, it significantly upregulated GABA levels in leaves and increased sodium accumulation in roots. The observed increase in Na^+^ content was associated with the overexpression of *NHX1*, encoding the tonoplast H^+^/Na^+^ antiporter responsible for vacuolar sodium sequestration. The authors speculated that the protective effect of Spm is at least partially related to the GABA pathway [123].

Spraying tea plants with Put significantly elevated the GABA content in the leaves [113]. In contrast, the Glu level remained unchanged. These effects were related to the increased expression of the *CsCuAO1* and *CsCuAO3* genes, encoding DAO. On the other hand, the Put application had no effect on the *CsGAD1-3* and *CsAMADH1* transcription levels [113]. Studies using three *Citrus* species, including *C. reticulata*, *C. sinensis*, and *C. paradisi*, showed that the Put treatment of plants increased GABA levels in leaves subjected to cold stress [124]. Exogenous Put induced low-temperature tolerance by reducing H_2_O_2_ production, enhancing flavonoid and phenol content, and increasing antioxidant activity.

### 4.3. Effect of Exogenous GABA on PA Pathways

The treatment of plants with GABA can improve stress resistance by affecting PA metabolism, Figure 3. It was found that under drought conditions, the application of GABA increases the accumulation of endogenous GABA and Glu in white clover leaves [125]. This was associated with the regulation of GABA shunt enzymes. Moreover, GABA stimulated the activity of enzymes responsible for PA biosynthesis and inhibited the function of DAO and PAO, suppressing PA degradation. As a result, higher levels of various types of PA, especially free Put, were observed. The beneficial effects of GABA on stress-induced damage, including improved water content and lowered lipid peroxidation and electrolyte leakage, were reversed by the addition of AG or 4N (GAD inhibitor), confirming the important role of this molecule in drought tolerance [125].

Exogenous GABA was used to mitigate the inhibition of muskmelon seedling growth caused by both Ca(NO_3_)_2_ [126] and salinity–alkalinity [94]. Under Ca(NO_3_)_2_ stress, the application of GABA regulated its own accumulation in different ways, upregulating it in leaves (in parallel with increased GAD and GABA-T activity) and reducing it in roots (inhibiting GAD activity and enhancing GABA-T activity). On the other hand, after GABA treatment, similar changes in PA levels occurred in both stressed organs, demonstrating a decrease in free Put and an increase in free Spm and Spd. The activities of the enzymes involved in PA synthesis were significantly stimulated by GABA, whereas DAO was downregulated in leaves and roots. The authors suggested that GABA acts through PAs, enhancing their production, reducing catabolism, and converting free Put to an insoluble bound form, to improve stress resistance [126]. The application of GABA to salinity–alkalinity-exposed muskmelon plants resulted in the restoration of Na^+^/K^+^ homeostasis and the amelioration of lipid peroxidation. GABA induced the accumulation of Put, Spd, and Spm and increased the expression of genes related to PA synthesis. When Spd production was inhibited with DCHA (dicyclohexylammonium sulfate), GABA-triggered positive effects were diminished. In addition, the treatment of plants with exogenous Spd had a similar beneficial impact on plants as GABA. Therefore, under salinity–alkalinity conditions, such as in the presence of Ca(NO_3_)_2_, PAs may be involved in GABA-dependent stress tolerance in muskmelon [94].

Li et al. [127] indicated that the addition of GABA protects creeping bentgrass against the adverse effects of various stress factors by promoting the accumulation of different groups of metabolites. GABA pretreatment improved several plant growth parameters, including photosynthesis, water content, and chlorophyll concentration, in the leaves of creeping bentgrass subjected to heat, drought, and salinity. Under all the stress conditions analyzed, GABA promoted the accumulation of Spd and total endogenous PAs, and increased the content of endogenous GABA, Glu, and Ala, involved in the GABA shunt, as well as Phe, Gly, and Asp. Moreover, after GABA treatment, the Put level was specifically enhanced under drought stress, while the Spm level was increased under heat stress. Differences in GABA-induced metabolomic changes with respect to amino acids and sugars were also observed between plants exposed to individual stress factors. The accumulation of fructose and glucose appears to be associated with GABA-dependent high-temperature tolerance, Pro and mannose with water deficit tolerance, whereas Arg, xylose, and trehalose with resistance to salt stress [127].

Hypoxia causes a significant increase in GABA content and GAD activity, as demonstrated in melon roots [128]. After treating plants with GABA, endogenous GABA and Glu levels further increased, while GAD activity decreased in stressed roots. The application of GABA affected PA metabolism. Under hypoxia, it induced the gene expression and activity of enzymes that participate in PA biosynthesis and reduced those responsible for PA catabolism (DAO and PAO). At the same time, an enhancement in free, soluble-conjugated, and insoluble bound PAs, including Put, Spm, and Spd, was observed. The results indicate that GABA confers hypoxia tolerance in melon seedlings by promoting PA production and suppressing PA oxidation, thus increasing their accumulation [128]. In contrast, low temperature was found to decrease endogenous GABA in tea plants [129]. The proteomic analyses of tea have shown that the application of GABA affects the level of Glu, PAs, and anthocyanins, modulating many metabolic pathways (including carbon fixation, nitrogen metabolism, flavonoid metabolism, TCA cycle, and others) and improving cold tolerance. However, the activities of GAD, GABA-T, and DAO as well as Put and Spm contents did not change significantly in GABA-treated plants. On the other hand, under low-temperature conditions, Spd accumulation was upregulated and PAO activity was reduced by exogenous GABA [129].

GABA is involved in the plant adaptation to heavy metal stress. GABA application to cadmium (Cd)-exposed maize plays a protective role against metal-induced toxic effects by ameliorating plant growth and photosynthesis, inhibiting Cd uptake and translocation, and increasing antioxidant defense [130]. Under stress conditions, GABA modified exogenous PA concentration and conversion. It induced a decline in the levels of free Put and insoluble bound Put, Spd, and Spm while increasing the accumulation of soluble conjugated Put and free and soluble conjugated Spd. The addition of GABA to Cd-treated plants resulted in a decreased expression of the genes related to PA biosynthesis (*ORDC* and *SPDS*) and oxidation (*PAO*). However, the transcript levels of the *ORDC* and *SPDS* genes were significantly higher compared to the control conditions. Thus, it has once again been confirmed that GABA participates in stress reactions by acting through PA [130]. The relationship between GABA application and PA biosynthesis was found to be essential in rice tolerance to arsenite (As^III^) [131]. Under stress, exogenous GABA reduced the content of Put, Spm, and Spd in both the roots and the shoots. On the other hand, this molecule increased PA accumulation in the roots of plants grown under control conditions. The treatment of As-exposed plants with GABA resulted in the upregulated expression of PA biosynthesis genes. Moreover, these plants showed increased expression of *PAO* and *DAO*, indicating enhanced PA catabolism. In parallel with modulated PA metabolism, an increase in the level of unsaturated fatty acids, amino acids, and the activation of antioxidant systems was observed in GABA-treated plants.

### 4.4. PA and GABA Transport Share Common Elements

It has been suggested that among the various groups of PA transporters, BAT proteins may function as PA exporters [132]. In *Arabidopsis*, BAT1 was originally identified as a GABA transporter, showing homology to the GABAP of *Aspergillus nidulans* and *Saccharomyces cerevisiae* [133]. However, detailed analyses indicated that it acts as a bidirectional transporter responsible for the export of amino acids from the cell across the plasma membrane. Moreover, when expressed in yeast cells, it was capable of transferring Ala, Arg, Glu, and Lys, while no transport activity for GABA was observed [133]. Using *Escherichia coli* as a model organism, subsequent studies confirmed that *Arabidopsis* AtBAT1.1, AtBAT1.2, and rice OsBAT1 function as both amino acid and PA antiporters. Additionally, transient expression in *Nicotiana benthamiana* revealed their localization [134]. The heterologous expression of *AtBAT1* in *E. coli* mutant, defective in Arg and PA exchangers, and competition analyses showed that this protein can transport Arg, Put, Spm, GABA, Glu, and Ala in a proton-driven manner [135]. Although knowledge about BAT proteins is still limited, the transport of PA and GABA via the same antiporter provides an additional opportunity to combine the functions of both types of molecules.

## 5. Crosstalk between GABA and Gasotrasmitters

Gasotrasmitters, including nitric oxide (NO), carbon monoxide (CO), and hydrogen sulfide (H_2_S), as well as hydrogen gas (H_2_) and methane (CH_4_), act as signaling molecules that participate in many developmental and cellular processes in plants. Furthermore, these gaseous particles are involved in plant defense mechanisms, which can enhance plant resistance to unfavorable environmental factors, both biotic and abiotic [136]. Among them, NO functions and NO-dependent pathways seem to be the best studied. Some evidence suggests that the crosstalk between GABA and NO may be beneficial for plants and essential for plant stress tolerance strategies [13].

### 5.1. NO Pathway

It was found that the application of NO or GABA enhances plant growth under stressful conditions [137]. Foliar sprays with SNP (sodium nitroprusside, a NO donor) or GABA alleviated the adverse effects of salinity in four perennial ryegrass cultivars by improving the maximum quantum efficiency of photosystem II (Fv/Fm) and decreasing sodium accumulation in leaves [138]. In white clover, exogenous NO (SNP application) and GABA were used to mitigate water deficit-induced damage [139]. A metabolomic analysis revealed that NO and GABA activate both common and different metabolic pathways in leaves, triggering a defense response. They increased the content of similar amino acids, sugars, organic acids, and sugar alcohols. On the other hand, citric acid accumulation was shown to be related to SNP application, while increased glycine, methionine, and aconitic acid levels were induced by exogenous GABA [139]. This suggests that both signaling molecules can act independently or dependently on each other.

**Figure 4:**
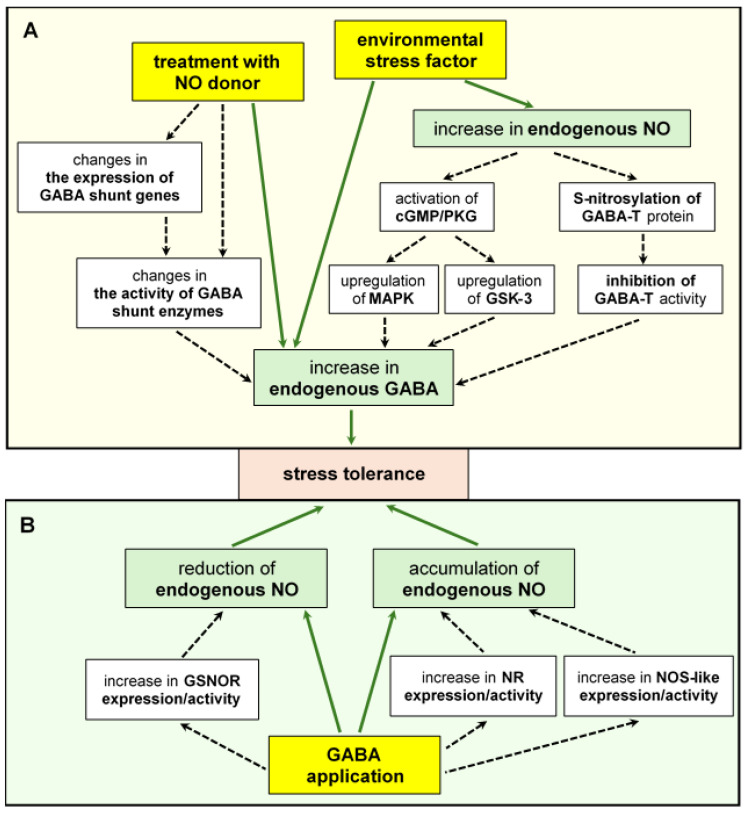
Crosstalk between GABA and NO in plant cells. NO has been shown to modify the GABA pathway (**A**), and GABA has been found to affect NO signaling (**B**). NO can change GABA production by acting through cyclic GMP and protein kinase G (PKG), upregulating mitogen-activated protein kinase (MAPK) gene and protein expression, or stimulating glycogen synthase kinase-3 (GSK-3) activity. Moreover, the NO-dependent S-nitrosylation of GABA aminotransferase (GABA-T) was shown to enhance the GABA level. GABA affects the endogenous NO level, increasing the activity and gene expression of nitrate reductase (NR), NO synthase (NOS)-like, and S-nitrosoglutathione reductase (GSNOR); the external sources of GABA and NO are highlighted in yellow, and the changes in their endogenous accumulation in green; the green arrows indicate the direction of the main changes/relationships identified in the studies, while the black dashed arrows the possibility of additional relations.

GABA-induced NO generation appears to play a particular role in enhancing plant stress tolerance, Figure 4. It is well known that in plant cells, various routes, both reductive and oxidative, participate in the production of NO. These include NR and NOS-like activity similar to animal NO synthase (NOS) [140]. *Arabidopsis* NOS1 (also known as NOA1, NO-associated protein) was originally identified as a NO-generating enzyme using arginine as a substrate [141]. Although later studies have shown that it functions as a GTPase [142], NO production related to NOS1/NOA1 has been demonstrated in plants subjected to stress conditions [143].

GABA was found to mediate NO synthesis pathways dependent on both NR and NOS-like activity. In soybean sprouts, it has been shown that GABA increased while 3-MP (3-mercaptopropionate, GABA synthesis inhibitor) decreased NO content under salinity conditions [144]. Elevated NO level promoted the biosynthesis of phenolic compounds and this was diminished by NO synthesis inhibitors, L-NAME (N-nitro-L-arginine methyl ester, NOS inhibitor) and sodium tungstate (NR inhibitor). GABA treatment reduced the inhibitory effects of 3-MP and the L-NAME on NO production. The results indicated that both GABA and NO increase the accumulation of phenolic compounds during soybean germination, and GABA acts through the NO pathway [144]. Under salinity–alkalinity stress, GABA pretreatment increased NO accumulation in muskmelon seedlings, activating the antioxidant response, regulating Na^+^/K^+^ balance, and improving plant growth [145]. This increase was related to the stimulation of NR activity and NOS-like activity. Moreover, the expression of the *NR* genes was significantly higher in plants exposed to GABA. On the other hand, c-PTIO (2-(4-carboxyphenyl)-4,4,5,5-tetramethylimidazoline-1-oxy-3-oxide, NO scavenger) did not change tissue GABA content, but diminished the promoting effects of GABA, suggesting that NO function as a downstream messenger in GABA-induced signaling [144]. The role of GABA in controlling NO production was analyzed in creeping bentgrass subjected to water stress [146]. GABA was found to affect nitrogen metabolism. It increased the NO level in the leaves of plants exposed to water deficit, activating both the NR and NOA protein. The GABA effect was related to a higher activity of antioxidant enzymes resulting in less ROS-induced damage [146]. Similarly, exogenous GABA improved growth and photosynthesis parameters, ameliorated nitrogen and sulfur assimilation, and activated antioxidant defense in wheat plants growing under salt stress [147]. GABA application increased NO accumulation in salt-treated plants compared to control plants. Using c-PTIO, it was shown that the beneficial effect of GABA was mediated by NO. Therefore, the authors concluded that under salinity conditions, exogenous GABA amplifies NO signaling to enhance stress tolerance [147]. The positive effects of GABA on NR activity/protein level/gene expression and nitrogen metabolism were also demonstrated in *Arabidopsis* seedlings [148], poplar seedlings under nitrogen deficiency [149], and the leaves of pakchoi under nitrogen-rich conditions [150], as well as in mung bean [151] and maize [152] plants subjected to salt stress.

The relationship between GABA and NO in alleviating metal toxicity was analyzed in tomato and brinjal seedlings exposed to arsenate (As^V^) [13,153]. Both GABA and SNP individually mitigated the negative effects of metalloid, improving plant growth, photosynthetic parameters, and oxidative defense, as well as upregulating sulfur assimilation (cysteine level) and Pro metabolism. The SNP-induced effects were reversed by the addition of cPTIO. The inhibition of NO synthesis (the treatment of vegetables with L-NAME) led to an intensification of As toxicity symptoms in the presence of GABA. This indicates that NO is essential for GABA-dependent metal stress tolerance. In contrast, GABA does not appear to be involved in the NO-dependent improvement of plant survival under As stress [13].

Exogenous GABA was found to trigger the resistance of cherry tomatoes to the fungus *Botrytis cinereal* [154]. This effect was associated with changes in endogenous NO. NO levels increased in GABA-treated fruits and leaves before inoculation with the pathogen and decreased after the inoculation process. Moreover, in inoculated fruit, GABA stimulated NR activity and, to a greater extent, S-nitrosoglutathione reductase (GSNOR, NO metabolizing enzyme using GSNO as a substrate) activity and gene expression. In contrast, no GABA-dependent changes were found in the expression of *Glb1* (non-symbiotic phytoglobin 1) involved in NO regulation during plant–microbe interactions. The essential role of GSNOR in the increase in GABA-dependent disease resistance was confirmed using its scavenger, N6022, and transgenic tomato plants overexpressing and suppressing *GSNOR*. GSNOR inhibition resulted in enhanced NO accumulation and greater fruit susceptibility to *B. cinereal*. The authors concluded that GABA, by regulating GSNOR, may help maintain NO levels at a stable, appropriate level after tomato inoculation with the pathogen [154].

On the other hand, NO may act upstream of the GABA signal, Figure 4. The treatment of bamboo shoots with SNP resulted in the accumulation of GABA (as well as PA and Pro) [155]. Exogenous NO stimulated NOS-like activity and modulated the activity of GAD and GABA-T involved in GABA synthesis and catabolism, respectively. As a result, an enhanced tolerance to chilling was observed. The authors suggested that SNP application and NO signaling can prevent cold damage when storing bamboo shoots at low temperatures [155]. Similarly, NO was shown to improve the cold resistance in tea (*Camellia sinensis*). When SNAP (S-nitroso-N-acetylpenicillamine, a NO donor) was applied to plants, both the activity and gene expression of GAD and GABA-T were modified in roots exposed to low temperatures [94]. However, the observed changes in enzyme activities were not correlated with the tissue GABA content, and no increase in its accumulation was found. Using cPTIO and L-NNA (Nw-nitro-L-arginine, NOS inhibitor) to reduce NO level, it was suggested that NO could modulate the GABA shunt, increasing GABA utilization during low-temperature storage [90].

Zafari et al. [156] used WT and transgenic tobacco plants with the overexpression and knockdown of the alternative oxidase (*AOX*) gene to verify whether the modified NO generation rate affects the gene expression of two amino acid-related routes, the GABA pathway and the phosphorylated pathway of serine biosynthesis (PPSB). During hypoxia, plants overexpressing *AOX* showed the highest leaf NO emission, while *AOX* knockdown plants exhibited the lowest. Increased NO production was correlated with enhanced NR activity and gene expression. Similarly, the highest level of *GSNOR* transcript was found in plants with *AOX* overexpression. *GAD* and *GABA-T* transcription levels did not change significantly in the first hours of stress, but they appeared to increase in overexpressors at a later stage of hypoxia. In contrast, an earlier activation of PPSB was observed, suggesting that this pathway, rather than the GABA shunt, plays a major role in maintaining nitrogen, carbon, and energy metabolism in plants subjected to an oxygen deficit and it is associated with NO metabolism [156]. In turn, Montilla-Bascón et al. [157] investigated the role of NO in barley tolerance to drought stress. Using transgenic plants with *Hb1* (non-symbiotic hemoglobin) overexpression, characterized by a lower NO content, it was shown that reducing NO biosynthesis results in enhanced plant resistance. The analysis of amino acid levels indicated that after exposure to drought, the GABA content increased in WT plants while it decreased in transgenic plants, similarly to Lys and Pro, suggesting a positive correlation between NO and GABA. However, the total content of PA was significantly higher in plants overexpressing *Hb1* that grow under water deficit, which was related to the accumulation of their precursors. The results suggest that NO may be involved in the regulation of PA biosynthesis and consequently, in drought tolerance [157].

NO signal transduction involves secondary messengers. These include cGMP (3′,5′-cyclic monophosphate) and guanylate cyclase (GC), the role of which in NO signaling pathways has been confirmed in animal cells [158]. It has also been suggested that cGMP can mediate NO-dependent GABA accumulation [159]. The involvement of NO in the UV-B stress-induced increase in GABA level was demonstrated in soybean sprouts [159]. UV-B-dependent GABA accumulation was reduced by the GC inhibitor (LY83583) and the protein kinase G (PKG) inhibitor (KT5823). These inhibitory effects were abolished by adding 8-Br-cGMP, a cGMP analog, suggesting that NO acts through cGMP/PKG. Moreover, under UV-B stress, NO, cGMP, and PKG mediated the upregulation of the MAPK (mitogen-activated protein kinase) gene and protein expression whereas MAPK was involved in increasing the GABA level [159]. In addition, a NO- and GABA-dependent increase in isoflavone content, associated with the stimulation of gene and protein expression, as well as the activity of chalcone synthase and isoflavone synthase, was observed during UV-B treatment. In the presence of the GSK-3 (glycogen synthase kinase-3) inhibitor, Bikinin, the UV-B-induced effects on GABA accumulation and GABA-related metabolic changes were weakened [160]). The obtained results clearly indicate that in soybean sprouts exposed to UV-B stress, both MAPK and GSK-3 can act as downstream signals in the NO/cGMP/PKG pathway, regulating GABA production by enhancing the gene, protein, and activity levels of the enzymes involved in GABA synthesis, DAO and AMADH [159,160].

One of the main mechanisms of NO action is believed to be the direct modification of the thiol groups in the cysteine residues. Through this reversible post-translational modification of proteins, called S-nitrosylation, NO can influence biological processes [161]. Using RNA interference, *GSNOR* knockdown tomato plants, with excessive NO accumulation, were created. By analyzing the site-specific nitrosoproteomic database, a conserved S-nitrosylation site was identified within the enzymes of the GABA-T family [162]. It was shown that under salinity–alkalinity stress, an increased NO level results in the S-nitrosylation of Cys316, Cys258, and Cys316 in SlGABA-TP1, TP2 and TP3, respectively. The substitution of Cys residues with Ser prevents the S-nitrosylation of the enzyme [163]. Cysteine modifications led to the inhibition of GABA-T activity, especially SlGABA-TP1, and an increase in GABA levels in tomato roots. Excess NO-mediated GABA was found to interact with the aluminum-activated plasma membrane malate transporter SlALMT14 and, as a consequence, reduced malate efflux occurred, decreasing stress tolerance. On the other hand, in tomato fruit, the tonoplast SlALMT9 appears to be a target for GABA. Under salinity–alkalinity, moderate NO increased the expression of *SlGABA-TP1*, resulting in GABA decomposition, enhanced SlALMT9 activity, and vacuolar malate storage [163].

The relationship between NO generation and the defense response to a subsequent pathogen attack was studied in potatoes exposed to the four inducers of systemic acquired resistance, including GABA and its isomer BABA (beta-aminobutyric acid) [164]. The authors assumed that the reversible storage of NO-elicited S-nitrosothiols (SNOs) is an important component of plant defense priming. The application of GABA and BABA enhanced NO generation in potato leaves at relatively low levels. Both agents also increased the SNO pool in leaves, but in a different time-dependent manner—GABA after 3 h, while BABA after 24 h of treatment. On the other hand, they upregulated the expression of histone *H2B* in a similar way. Based on the obtained results, it was concluded that GABA and BABA can induce short-term NO-dependent imprint activation and facilitate acquired resistance after pathogen inoculation [164].

### 5.2. Other Gaseous Molecules

Little is known about the relationship between GABA and other gasotransmitters in plants. Relatively recently, H_2_S has been recognized as a signaling molecule involved in the control of processes occurring in plant cells, mainly due to its possibility of the reversible modification of oxidized cysteine residues to form persulfides [165]. More and more attention is paid to the use of H_2_S donors and scavengers to improve the growth parameters of plants exposed to unfavorable environmental conditions [166,167].

The treatment of persimmon fruit with NaHS, a H_2_S donor, or GABA resulted in a reduction in chilling-induced damage, manifested by fruit browning [168]. Both molecules activated antioxidant systems, including the enhanced activity of antioxidant enzymes and increased accumulation of ascorbic acid, which in turn lowered endogenous H_2_O_2_ and lipid peroxidation, and protected membrane integrity. Both GABA and H_2_S promoted the accumulation of phenols and flavonoids, and improved fruit quality during cold storage by reducing the activities of polygalacturonase and pectin methylesterase and suppressing softening [168]. This suggests that GABA and H_2_S can induce similar defense mechanisms. In banana fruit, exogenous H_2_S has been shown to alleviate chilling-induced injury by protecting chlorophyll from degradation, lowering ROS generation, and increasing Pro content [169]. As in persimmon, the application of H_2_S upregulated the activity of antioxidant enzymes and promoted the accumulation of phenolics, ascorbic acid, and glutathione. Additionally, an elevated GABA content, related to increased GAD and GABA-T activity, was observed in banana fruits treated with H_2_S, indicating that the interaction between these signals may be important for protection against low temperature during storage [169]. Similar studies have been carried out on fresh-cut peaches [170]. H_2_S fumigation was used to maintain fruit quality and prevent browning. Among others, increased levels of phenolics, some amino acids, and their derivatives, including GABA, Pro, and PAs, were found in fruits treated with H_2_S. The authors suggested that H_2_S-induced GABA accumulation, accompanied by enhanced GAD activity, plays a beneficial role in cutting stress [170].

Relations between GABA pathways and the signaling of other gaseous molecules have not yet been confirmed in plants, although CO, CH_4_, and H_2_ have been found to interact with various plant regulators and secondary messengers such as NO, H_2_S, H_2_O_2_, Ca^2+^, and phytohormones [139,171,172]. However, some results from animal studies suggest this possibility. The functional interplay between CO and NO-GABA signaling in the magnocellular neurosecretory cells of the hypothalamus paraventricular nucleus, which release the neurohormone vasopressin (VP), within which GABA is a dominant inhibitory neurotransmitter, was demonstrated in rats with heart failure, HF [173]. In HF rats, the enhanced gene and protein expressions of heme-oxygenase 1 and a related increase in CO synthesis occur in VP neurons, and excitatory CO signaling results in blunted NO and GABA inhibitory function. In overweight humans, H_2_ was shown to modulate the levels of Glu, glutamine, and GABA involved in appetite stimulation [174].

## 6. GABA-ROS Relationship

ROS are chemical compounds containing oxygen atoms with unpaired electrons (radicals) or O–O bonds that can participate in many reactions and play a significant role in the functioning of living organisms. Radicals include superoxide anion radical (O_2_^•−^), hydroperoxide radical (HO_2_^•^), and HO^•^. Free radicals are highly reactive and thus typically short-lived, often unable to leave the subcellular location of their generation [175]. However, ROS that are not radicals, including singlet oxygen (^1^O_2_), ozone (O_3_), and hydrogen peroxide (H_2_O_2_), are usually less reactive and thus capable of migrating to different sites, traversing cellular membranes [175]. ROS are widely considered to be harmful to plants and animals. They induce various detrimental reactions, such as damage to lipids, proteins, and nucleic acids [176]. The action of ROS on proteins can result in many modifications, including the oxidation of specific amino acid residues, fragmentation of the polypeptide chain, formation of cross-links, and aggregation or change in charge [177]. The main consequence of oxidative protein damage is their increased degradation by proteases [178]. In DNA, ROS reacts with nitrogenous bases and deoxyribose to induce a multitude of types of DNA disturbances. These include single- and double-strand breaks in the DNA helix, the formation of apurinic and apyrimidic sites, and base modifications. The rupture of nucleosomes results in the fragmentation of DNA, which in turn leads to complications in the compaction and coiling of DNA within chromatin [179]. The hydroxyl radicals and singlet oxygen react with unsaturated fatty acids by removing a hydrogen atom, which results in the formation of lipid hydroxyperoxides. These compounds can be degraded into a variety of products, including aldehydes and alcohols. Furthermore, in the presence of a metal catalyst, they participate in the Fenton reaction, which leads to the formation of reactive alkoxy radicals (RO^•^). HO^•^, therefore, initiates a chain reaction that causes lipid peroxidation [179,180]. ROS-induced damage to macromolecules results in a significant disruption of metabolic pathways and processes [177].

**Figure 5:**
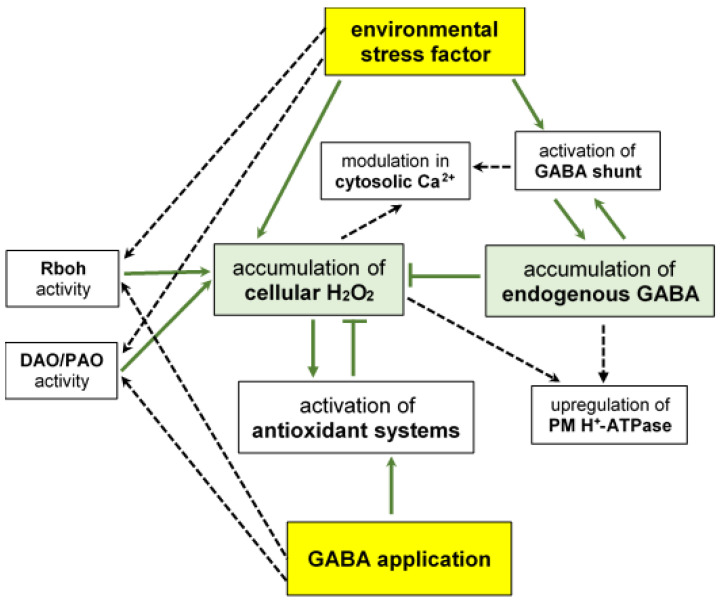
Interrelation between GABA and H_2_O_2_. GABA can affect H_2_O_2_ levels in two ways, by modulating H_2_O_2_ signaling and activating antioxidant systems. The increase in GABA levels, observed under stress conditions, occurs in parallel with a decrease in H_2_O_2_ accumulation. After the treatment of plants with GABA, the stimulation of antioxidant systems (both enzymatic and non-enzymatic) was found. On the other hand, exogenous GABA can enhance H_2_O_2_ production for signaling function by activating diamine oxidase (DAO) or polyamine oxidase (PAO) in the apoplast or plasma membrane-bound NADPH oxidase (Rboh). One of the aspects of the interaction between GABA and H_2_O_2_ appears to be the regulation of key proteins involved in plant tolerance, including the plasma membrane proton pump (PM H^+^-ATPase) and Ca^2+^ channels (the external sources of GABA and H_2_O_2_ are highlighted in yellow, and their endogenous accumulation in green; the green arrows indicate the direction of the main changes/relationships, activation or inhibition, identified in the studies, while the black dashed arrows the possibility of additional relations).

Hydrogen peroxide is a key ROS molecule generated within living cells from a multitude of endogenous sources. H_2_O_2_ is not always considered to be inherently harmful. The presence and action of hydrogen peroxide in plants is a multifaceted phenomenon [181]. H_2_O_2_ has been demonstrated to possess both beneficial and detrimental properties. At high concentrations, it has been shown to exhibit characteristics similar to those of other ROS, causing damage to biological systems. In contrast, at low concentrations, it has been identified as a signaling molecule that triggers defense mechanisms in plants growing under unfavorable environmental conditions [181,182,183]. H_2_O_2_ is considered a necessary evil [176]. For this reason, it is essential to maintain a balance between the beneficial and deleterious effects induced by H_2_O_2_ to ensure optimal cellular function. H₂O₂ can be transported by aquaporins that are localized in the cell membranes, thereby participating in the regulation of cell signaling [184]. H₂O₂ differs from other ROS in its relatively long half-life. It can be produced in different cell compartments including chloroplasts, mitochondria, peroxisomes, endoplasmic reticulum (ER), and apoplast [176,185]. The main sources of hydrogen peroxide are the disruption of the electron transport chain in chloroplasts and mitochondria, plasma membrane NADPH oxidase (Rboh), and cell wall-associated peroxidases [186], Figure 5. NADPH oxidases initially generate a superoxide anion radical, rapidly dismutated into H₂O₂ by SOD [176]. Furthermore, PA oxidases such as DAO and PAO, involved in PA degradation pathways, also serve as a source of H₂O₂ [187]. It is well known that ROS production increases significantly in plant responses to stressful conditions. The endogenous H₂O₂ content is typically much higher in plant cells than in animal cells. Plant cells can survive with H₂O₂ levels that are lethal to animal cells. This plant tolerance is linked to highly efficient antioxidant systems. ROS scavenging mechanisms can be classified into two categories: enzymatic and non-enzymatic antioxidant systems that function collectively to inactivate free radicals. Enzymatic systems include mainly SOD, ascorbate peroxidase (APX), catalase (CAT), glutathione reductase (GR), and glutathione peroxidase (GPX) [188]. Non-enzymatic systems operate using low-molecular-weight antioxidants, including glutathione, ascorbic acid, and flavonoids, which have been recognized for their ability to neutralize hydroxyl radicals and singlet oxygen [188].

When plants are exposed to stress conditions, the balance between ROS production and the efficiency of antioxidant defense systems is impaired, resulting in excessive ROS accumulation and inducing oxidative stress. Many reports indicated that GABA helps plants survive under unfavorable conditions by stimulating antioxidant systems, Figure 5 [14,189,190,191,192,193,194].

Salt significantly delayed the seed germination and growth of lettuce (*Lactuca sativa* L.). It was observed that the application of GABA eliminated salt-induced negative effects on these processes. First, in stressed plants, it improved photosynthetic parameters, including an increase in Fv/Fm and the electron transport flux by photosynthetic electron carriers in thylakoid membranes, and a decrease in non-photochemical quenching (NPQ). Second, GABA contributed to the increased activity of CAT, APX, and SOD in salt-exposed plants [190]. A similar stimulating effect of GABA on the antioxidant system was observed in maize seedlings and mung bean plants subjected to salt stress [151,194]. Other studies have shown that anthurium cut flowers can be damaged at low temperatures due to reduced membrane fluidity and H_2_O_2_ accumulation. The treatment of anthurium with GABA (applied by pre-harvest spraying or post-harvest stem end dipping) was found to help it survive storage at 4 °C. GABA treatment significantly delayed flower browning, increased the ratio of unsaturated to saturated fatty acids, and reduced H_2_O_2_ accumulation. The decrease in the H_2_O_2_ content was due to the increased activity of antioxidant enzymes: SOD, CAT, APX, and GR [191]. Additionally, the protective role of GABA was observed in tomato plants exposed to low temperatures. Similarly, as with other forms of stress, GABA has been found to significantly attenuate oxidative damage induced by low temperatures (6 h at 5 °C for 5 days), thus protecting the integrity of the cell membrane and reducing the levels of MDA and H₂O₂. This beneficial effect of GABA was linked to an increase in phenylalanine ammonia-lyase (PAL), CAT, SOD and APX activity [193]. A similar phenomenon was observed in *Capsicum annuum* seedlings subjected to low light stress after GABA treatment. Under stress conditions, the photosynthetic parameters of *C. annuum* plants exhibited a decline, accompanied by a notable increase in superoxide anion and hydrogen peroxide production. On the contrary, plants subjected to both GABA and stress exhibited enhanced photosynthetic parameters and diminished ROS production. Furthermore, an increase in the activity of numerous antioxidant system components, including SOD, CAT, APX, GPX, monodehydroascorbate reductase (MDAR), dehydroascorbate reductase (DHAR), GR, ascorbate, and glutathione, was observed in these plants. The obtained results suggest that GABA mitigates low-light stress by modulating the antioxidant defense system [192]. The contamination of the environment with polycyclic aromatic hydrocarbons (PAHs), such as phenanthrene, has been shown to exert a detrimental impact not only on human health but also on the growth and functionality of plants. The application of phenanthrene to plants was found to increase the production of ROS. It has been demonstrated that the simultaneous treatment of plants with GABA and phenanthrene results in a reduction in ROS production through the increased transcription and activity of APX, CAT, and SOD. Additionally, GABA elevated glutathione levels by stimulating the expression of its biosynthetic genes, specifically *GSH1* and *GSH2*. However, treatment with buthionone sulfoximine (a glutathione biosynthesis inhibitor) prevented the GABA-induced response in plants exposed to phenanthrene. This outcome indicates that GABA mitigates phenanthrene-induced stress through a glutathione-dependent mechanism [195].

Under salt stress, H_2_O_2_ production was shown to be effectively inhibited by exogenously applied GABA in *Caragana intermedia* [2]. The addition of GABA simultaneously with NaCl caused an increase in the expression of many genes, including those encoding proteins involved in producing H_2_O_2_, such as Rboh or DAO. This is interesting because GABA contributes both to the production of H_2_O_2_ in the initial stress phase when hydrogen peroxide is required as a signaling molecule to trigger defense responses, and later to the elimination of excess ROS by activating antioxidant systems [2]. H_2_O_2_ can activate various significant proteins, such as the plasma membrane proton pump (PM H^+^-ATPase), a key enzyme in plant adaptation to stress conditions and important for ion transport across the plasma membrane [196,197]. The role of the interaction between ROS and PAs in the regulation of ion transport across the plasma membrane in the context of plant adaptation to stress was presented by Pottosin et al. [198]. In response to hypoxic conditions, plants exhibit a notable elevation in the levels of GABA within their tissues. Hypoxia-induced increase in GABA content is essential for the restoration of membrane potential and prevention of the ROS-induced disruption of cytosolic K^+^ homeostasis and Ca^2+^ signaling. Hypoxia causes H₂O₂ accumulation in the cell, which activates ROS-induced Ca²⁺ uptake channels and further exacerbates K⁺ loss through non-selective cation channels. However, GABA elevation restores membrane potential via the pH-dependent regulation of PM H⁺-ATPase and generates more energy through the activation of the GABA shunt pathway and the TCA cycle [199]. Under oxidative stress, the activity of the GABA shunt is of particular importance, as it functions as an electron donor in the electron transport chain within mitochondria [200].

Studies on *Arabidopsis* mutants have demonstrated that *pop2-5*, overaccumulating GABA, exhibits enhanced tolerance to salt stress. This tolerance was the result of the increased activity and gene expression of PM-H^+^-ATPase (*AHA2*), higher gene expression of sodium-proton antiporters (*SOS1* and *NHX1*), and a lower level of H_2_O_2_ production in tissues. H_2_O_2_ formation was significantly elevated in *gad1,2* mutants, with reduced GABA levels compared to *pop2-5*, as a consequence of salt stress [11]. This indicates that the GABA shunt components play an important role in preventing ROS accumulation [201]. Other studies have shown that *ssadh* mutants (*Arabidopsis* T-DNA knockout mutants of SSADH) are sensitive to heat and UV. They develop necrosis when exposed to these stresses. Furthermore, both UV and heat cause a rapid increase in hydrogen peroxide levels in *ssadh* mutants [202]. Similarly, *Arabidopsis* GABA-T mutants (*pop2-1*, which is a transient mutant, and *pop2-3*, which is a T-DNA insertion mutant) with markedly reduced GABA levels showed premature leaf senescence under various stress conditions [203]. The *pop2-1* and *pop2-3* mutations reduced photosynthetic efficiency and chlorophyll content, as well as increased membrane ion leakage in leaves and elevated H_2_O_2_ levels [203]. The presented data demonstrate that in *Arabidopsis*, mutations in the GABA-T coding genes elicit disparate responses to stressors. The *pop2-5* mutant exhibited enhanced tolerance to salt stress, which was related to increased GABA levels and diminished H₂O₂ accumulation. On the contrary, the *pop2-1* and *pop2-3* mutants exhibited elevated hydrogen peroxide levels and reduced GABA content in plants subjected to various stressors, including wounding, dehydration, darkness, and cold.

The GABA shunt was found to play an important role in maintaining ROS homeostasis in *Arabidopsis* guard cells [204]. It was observed that the disruption of GAD2 (*gad2* mutant) significantly reduced GABA accumulation in leaves, increased stomatal conductance, and caused greater susceptibility to drought conditions than compared to WT seedlings. On the other hand, additional mutations in the other GAD-encoding genes (*GAD1*, *GAD4*, and *GAD5*) and the generation of the *gad1/2/4/5* mutant resulted in a more pronounced reduction in GABA levels in plants. Moreover, in the quadruple *gad1/2/4/5* mutant, a loss of the more open stomatal pore phenotype, observed in the *gad2* mutant, was found. This mutant showed increased Ca²⁺ concentrations in guard cells compared to both wild-type and *gad2*. The results indicate that the resting cytosolic Ca²⁺ concentration is affected by the profound impairment of GABA synthesis, observed in the *gad1/2/4/5* mutant. Furthermore, severe impairment of GABA synthesis was shown to cause significant ROS accumulation in the guard cells of the *gad1/2/4/5* mutant compared to the cells of WT and *gad2* leaves. The restriction of apoplastic Ca^2+^ by EGTA treatment reduced ROS accumulation in guard cells and increased stomatal conductance in the quadruple mutant. This suggests a key role of GABA shunt components in ROS homeostasis, involving a calcium ion-mediated pathway [204].

## 7. Melatonin and GABA Signaling

Melatonin (N-acetyl-5-methoxytryptamine) is a chemical compound well known from the animal kingdom, discovered as a pineal hormone in ox in 1958 [205]. However, it is a highly conserved molecule synthesized in many groups of organisms, from bacteria and green algae to higher plants [206,207]. In both the animal and plant kingdoms, the precursor to its synthesis is the protein amino acid tryptophan [208,209]. From a chemical point of view, melatonin acts as an antioxidant that can interact with ROS and RNS (reactive nitrogen species), and one molecule of melatonin is able to scavenge four to ten molecules of ROS or RNS [210]. In addition to its unquestionably evolutionarily fundamental role as a free radical scavenger, in plants, melatonin acts as a regulator of basic processes such as growth [211,212], root organogenesis [213], flowering [214], and others. Melatonin plays an important role in nitrogen uptake and assimilation [215]. Many studies have indicated that the treatment of plants with melatonin results in the altered expression of various genes, which affects growth processes [209,216]. Furthermore, this compound has been implicated in plant responses to abiotic stresses including salt [217], drought [212], oxidative stress [218], and cold [219]. Under biotic stress conditions, the exogenous application of melatonin leads to reduced disease symptoms in some bacterial or fungal infections of plants [211]. Treatment with melatonin alleviates stress-induced damage mainly by reducing ROS accumulation [220,221]. Thus, melatonin acts similarly to GABA in plant cells [222]. Another common feature of these two growth regulators is their effect on nitrate metabolism [150]. This raises the question: is there a relationship between these molecules?

Many studies have shown that both melatonin and GABA, independently of each other, reduce the negative effects of stress. On the other hand, it appears that their effects can overlap. Under some stressful conditions, each of them independently has a beneficial effect on plant functioning, facilitating survival. However, when plants exposed to an adverse stressor were treated simultaneously with melatonin and GABA, the molecules acted synergistically. Their combined effect resulted in greater or novel benefits [223]. For example, under cadmium stress, the application of GABA together with melatonin significantly reduced the levels of Cd and malondialdehyde (MDA) in seeds by upregulating the activities of antioxidant enzymes, thereby alleviating the toxic effect of stress. In general, the combination strategy significantly improved tomato seed germination and its resistance to cadmium [223]. The positive effect of the interaction between melatonin and GABA was also observed by Shomali et al. [224]. They showed that the simultaneous treatment of *Vicia faba* with melatonin and GABA had a synergistic beneficial effect on photosynthesis. Under osmotic and salinity stress conditions, both melatonin and GABA, and their combined application, increased leaf area, number of flowers, dry and fresh weight of the shoots, and total biomass. Compared to the control, plants treated with GABA and melatonin developed larger stomata with a wider aperture. In such plants, Fv/Fm was higher, whereas NPQ was decreased in leaves exposed to the stresses.

Other studies have demonstrated that melatonin can mitigate the adverse effects of stress, with GABA playing an essential role in this process. The yield quality of kiwi plants is significantly diminished when they are subjected to flood-related stress [225]. The treatment of plants with exogenous melatonin was shown to improve plant functioning under waterlogging. Before the imposition of flood stress, the plants were irrigated with a melatonin solution. Relative electrolyte leakage and MDA content were elevated in control plants subjected to flooding. However, the application of melatonin resulted in a reduction in these parameters, indicating that flooding-induced stress has been alleviated. Additionally, melatonin treatment enhanced the activity of superoxide dismutase (SOD) and peroxidase enzymes in the roots of kiwifruit plants, thus reducing the accumulation of ROS and mitigating the toxic effects of ROS generated under the conditions of waterlogging stress. Furthermore, elevated levels of GABA and Pro have been observed in the roots of melatonin-treated kiwi plants [225]. The GABA content was also found to increase in response to waterlogging stress. However, melatonin-treated plants exhibited significantly higher GABA levels compared to those not pretreated.

It has been demonstrated that plant treatment with melatonin can result in a delay in the senescence process. Okra (*Abelmoschus esculentus* L.) is one of the vegetables that are particularly susceptible to deterioration during post-harvest storage. The application of melatonin to vegetables resulted in a delay in senescence and the maintenance of quality throughout the storage period. It was demonstrated that hormone levels were altered in the plants treated with melatonin. The IAA and GA contents increased, while the ABA levels decreased. Furthermore, melatonin treatment caused an increase in endogenous GABA in okra. The alterations in GABA levels related to melatonin treatment were attributed to the enhanced expression of the *AeGAD1* and *AeGAD2/3* genes [226]. Similarly, the expression of the genes encoding the proteins involved in GABA biosynthesis, *PpGAD1* and *PpGAD4*, was increased in yellow-fleshed peaches (*Prunus persica* L.) treated with melatonin. A marked accumulation of GABA was also observed in these plants. This increase was due not only to the upregulated expression of genes encoding GAD, but also to the reduced expression of the gene encoding GABA-T, *PpGABA-T*, which is responsible for the degradation of GABA [227]. Cao et al. [228] also observed an increase in GABA content when peach fruits were treated with melatonin. They showed that melatonin mitigates the effects of cold injury in plants by increasing the expression of *PpGAD*. Moreover, GABA accumulation was found in mango fruits previously treated with melatonin. This increase was correlated with a higher activity of the GABA shunt pathway [229]. In tomato fruits, Sharafi et al. [230] showed that melatonin treatment induced GABA shunt during cold storage. Melatonin was shown to increase the activity of the GAD, GABA-T, and SSADH enzymes.

## 8. Summary

Understanding the processes responsible for the regulation of GABA levels in plants is important for several reasons. Acting as an inhibitory neurotransmitter, GABA is essential for the proper functioning of the human body. Therefore, it is important to provide food, including plant-based food, rich in this compound. On the other hand, as a signaling molecule, GABA is involved in the control of plant growth and development, allowing them to adapt flexibly to the prevailing environmental conditions. Thus, the modulation of GABA content is useful for creating crop varieties resistant to stress factors.

GABA has been shown to interact with different secondary messengers that affect cellular metabolism and plant responses to stress. The crosstalk between GABA and all groups of plant hormones, including IAA, ABA, ethylene, JA, SA, GAs, CKs, BRs, and SLs, was confirmed. Both types of molecules regulate the processes of biosynthesis and the signal transduction of each other. Similarly, GABA action is related to phytohormone-like polyamines such as Put, Spm, and Spd. This relationship may be due, at least in part, to the polyamine-dependent GABA formation. One of the best-studied examples of GABA partners involved in cell signaling appears to be NO. NO has been found to function upstream and downstream of the GABA signal. NO may regulate GABA production directly through the reversible S-nitrosylation of biosynthetic enzymes or indirectly by activating protein kinases.

Although the knowledge of GABA interactions with other cellular regulators is constantly expanding, many aspects remain unclear. This concerns, among others, the connection between GABA and the messengers less studied, such as H_2_S or melatonin. Furthermore, the effect of H_2_O_2_ on the GABA pathway is worth elucidating, since it is well known that GABA can act by modulating ROS levels. It is still an open question whether H_2_S and/or H_2_O_2_ can, like NO, post-translationally modify the proteins involved in GABA signaling. Answers to these and similar questions will provide a more complete picture of the complex network of relationships between GABA and other molecules and their role in the regulation of the processes occurring in plant cells.

## Data Availability

No new data were created or analyzed in this study. Data sharing is not applicable to this article.
